# Açaí (*Euterpe oleracea* Mart.) in Health and Disease: A Critical Review

**DOI:** 10.3390/nu15040989

**Published:** 2023-02-16

**Authors:** Lucas Fornari Laurindo, Sandra Maria Barbalho, Adriano Cressoni Araújo, Elen Landgraf Guiguer, Arijit Mondal, Gabrielle Bachtel, Anupam Bishayee

**Affiliations:** 1 Department of Biochemistry and Pharmacology, School of Medicine, University of Marília, Marília 17525-902, SP, Brazil; 2 Department of Biochemistry and Pharmacology, School of Medicine, Faculdade de Medicina de Marília, Marília 17519-030, SP, Brazil; 3 Postgraduate Program in Structural and Functional Interactions in Rehabilitation, University of Marília, Marília 17525-902, SP, Brazil; 4 Department of Biochemistry and Nutrition, School of Food and Technology of Marília, Marília 17500-000, SP, Brazil; 5 Department of Pharmaceutical Chemistry, M.R. College of Pharmaceutical Sciences and Research, Balisha 743 234, India; 6 College of Osteopathic Medicine, Lake Erie College of Osteopathic Medicine, Bradenton, FL 34211, USA

**Keywords:** *Euterpe oleracea*, açaí, antioxidant, anti-inflammatory, antiproliferative, health benefits

## Abstract

The açaí palm (*Euterpe oleracea* Mart.), a species belonging to the *Arecaceae* family, has been cultivated for thousands of years in tropical Central and South America as a multipurpose dietary plant. The recent introduction of açaí fruit and its nutritional and healing qualities to regions outside its origin has rapidly expanded global demand for açaí berry. The health-promoting and disease-preventing properties of this plant are attributed to numerous bioactive phenolic compounds present in the leaf, pulp, fruit, skin, and seeds. The purpose of this review is to present an up-to-date, comprehensive, and critical evaluation of the health benefits of açaí and its phytochemicals with a special focus on cellular and molecular mechanisms of action. In vitro and in vivo studies showed that açaí possesses antioxidant and anti-inflammatory properties and exerts cardioprotective, gastroprotective, hepatoprotective, neuroprotective, renoprotective, antilipidemic, antidiabetic, and antineoplastic activities. Moreover, clinical trials have suggested that açaí can protect against metabolic stress induced by oxidation, inflammation, vascular abnormalities, and physical exertion. Due to its medicinal properties and the absence of undesirable effects, açaí shows a promising future in health promotion and disease prevention, in addition to a vast economic potential in the food and cosmetic industries.

## 1. Introduction

The açaí palm (*Euterpe oleracea* Mart.), a species belonging to the palm tree (*Arecaceae*) family, is native to several countries in the Amazon region of tropical Central and South America, including Brazil, Ecuador, and Venezuela [1]. Although açaí has been cultivated in its indigenous terrain for thousands of years as a multipurpose dietary plant, its recent introduction to regions outside its origin has rapidly expanded global demand for its fruit (açaí or açaí berry) in particular [2]. To meet the increasing rates of açaí consumption, Brazil has become its most important producer and exporter [3].

On a yearly basis, Brazil generates over 9 billion US dollars in açaí-based revenue [4,5,6]. The popularized use of açaí has warranted further scientific research on its botanical background, remarkable nutritional profile, and bioactive properties. The seed of açaí constitutes 80% to 95% of the overall proportions of the fruit [7]. At maturity, an individual açaí berry is 1.5–2.0 cm wide with black and purple coloration [4]. Thus, substantial amounts of açaí are necessary to provide adequate yield to meet the demands of consumption by the millions of people that rely on açaí as an important source of nutrients. For natives that live among the Amazon territory, especially those within the Brazilian states of Pará and Amapá, açaí has significant dietary and agricultural implications [8]. Pará is the predominant contributor to açaí production in Brazil [2,3]. However, due to increased global consumption of açaí, Pará natives no longer constitute the largest concentration of açaí consumers. The demand for açaí has grown considerably in southeastern and midwestern Brazilian populations as well [9]. As demonstrated by agroclimatic zoning studies, açaí crops, also known as açaí groves, have reached nonnative soils in other states of the country, such as Espírito Santo [3].

Açaí is both consumed and harvested daily by the residents of Brazil. Upon harvesting açaí, Amazonian locals sell the fruit to traders along the Amazon River [10]. Subsequently, traders wholesale the crop to market representatives. After processing the açaí into a frozen pulp, market representatives sell the frozen pulp or preserve it further for later use in the export market. The lengthy harvesting process results in time-sensitive nutrient depletion and may deem the fruit unfit for transportation to distant markets. Additionally, açaí palms grow exclusively in the Amazon biome [4]. Therefore, fresh açaí berries are unattainable outside of Brazil. The current literature lacks report of successful cultivation of açaí trees beyond the Amazon region. For this reason, the benefits of açaí transportability via frozen pulp production outweigh the time and energy involved in the process [11]. In the case of most açaí products, the pulp is mechanically extracted to yield a viscous juice. However, açaí can also be used to make ice cream, energy drinks, pharmaceuticals, and cosmetic products [6,12,13,14]. Because the antioxidant activity of açaí may be affected by processing and storage, it is essential to consider the preservation methods used to maintain the quality and activity of bioactive nutrients in açaí goods [15].

X-ray diffractometry studies have revealed that the morphology of polysaccharides, fatty acids, and proteins in freeze-dried açaí pulp appears as a spongy matrix with a partially crystalline molecular structure. Crystalline sugars have relatively low hygroscopicity. Powdered products with crystalline sugars demonstrate greater stability in various atmospheric environments [11]. Thus, freeze-drying methods are essential to the facilitation of access to açaí through the protection of its bioactive compounds and nutrients from degradation during transport [2].

The macronutrient composition of freeze-dried açaí pulp is relatively unique for a fruit. Lipids comprise half of the chemical profile of açaí pulp, and therefore, largely account for its classification as an energy-dense food. In comparison to other fruits, freeze-dried açaí pulp has a higher total dietary fiber content and lower total carbohydrate content [1,8]. Açaí pulp also contains a multitude of proteins, minerals (e.g., calcium, magnesium, potassium, manganese, copper, nickel, boron, chromium), and vitamins (e.g., B1, B6) [16]. Brazilian locals traditionally incorporate açaí into their daily diets via energy-dense smoothies or assorted fruit dishes [17].

Freeze-dried açaí has a sebaceous texture. A variety of fatty acids have been identified in assays of the nutritional constitution of açaí [18]. While unsaturated fatty acids exist in predominance, saturated forms (e.g., lauric acid, palmitoleic acid, palmitic acid, myristic acid) are also present [19]. The heart-healthy lipid profile of açaí, in conjunction with its high levels of antioxidants and fiber, substantiate its significance within the functional food industry as a health-promoting food. Furthermore, these data points elucidate the nomenclature of *E. oleracea*, as the term “oleracea” is derived from the word “oil.” The scientific name of this species not only describes its oily quality, but also alludes to its noteworthy lipid content [20].

**Figure 1:**
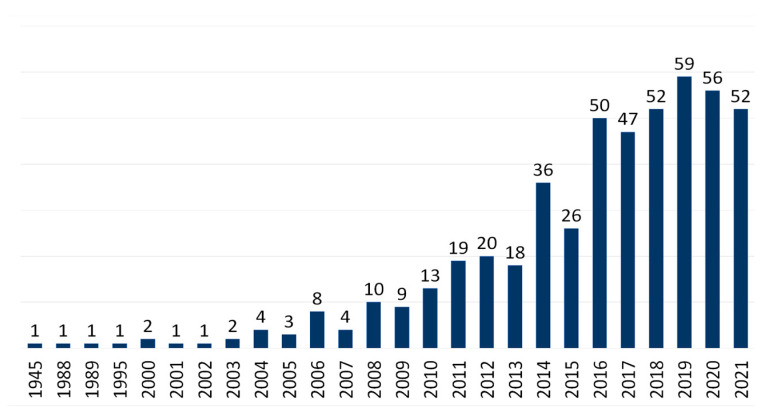
Increasing publications on *E. oleracea* indexed by PubMed since 2004.

It has been noted that açaí fruit extracts may have antioxidant and anti-inflammatory actions linked to the prevention and treatment of risk factors for diabetes, dyslipidemia, hypertension, and cardiovascular diseases (CVDs). Furthermore, açaí has been shown to exhibit anticancer, antiatherogenic, antimicrobial, antinociceptive, anticonvulsant, antileishmanial, and antiaging activities. Moreover, research has indicated the tissue-protective effects of açaí on several organs, such as the heart, liver, lungs, kidneys, and brain [21,22,23,24,25]. Publications on the effects of açaí on human health have increased with time (Figure 1). Nonetheless, only two reviews in the current literature have specifically addressed the health benefits of this fruit. One review substantiated the chemoprotective effects and safety of açaí [26]. The other review focused on the effects of açaí on overall health [21]. Additional in vitro, in vivo, and clinical studies on the health implications of açaí have since been published as more of its bioproperties (e.g., cardioprotective, hepatoprotective, renoprotective, antihypertensive, antilipidemic, and antidiabetic effects) continue to be discovered. Collectively, the present literature on açaí describes its prospective preventive and therapeutic capacity in the setting of various health conditions but lacks the most recent data on its health benefits. Therefore, this study aims to perform an up-to-date, comprehensive, and critical review of the biological and pharmacological activities of açaí-derived products and constituents linked to the health benefits of açaí, as well as their related cellular and molecular mechanisms of action.

## 2. Botanical Aspects

**Figure 2:**
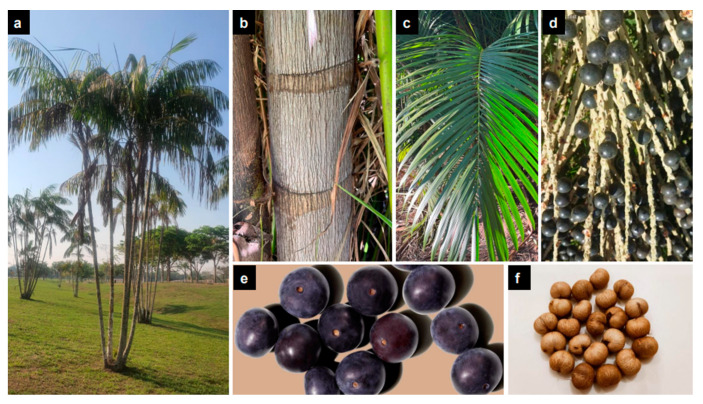
Photographs of various parts of *E. oleracea.* (**a**) whole plant; (**b**) stem; (**c**) leaf; (**d**) panicles of fruits; (**e**) isolated fruits; and (**f**) isolated seeds.

Açaí, popularly known as açaí-do-Pará, açaizeiro, or açaí-de-toceira, is a palm tree (Figure 2a) native to the Amazon Basin [10]. Açaí palms have stems (Figure 2b) that can reach 30 m in height and 18 cm in diameter. These trees predominantly mature in a multi-stem pattern and can reach up to 45 stems in the adult stage of their development. At the base of each stipe, reddish, dense, superficial, and fasciculate roots with aerenchymas and lenticels create an aggregate network 30 to 40 cm above ground [27]. Açaí stems tend to be cylindrical, ringed, and erect. Scars from the senescent leaves (Figure 2c) often form nodes and internodes along the açaí stem [28]. Additionally, the bunch-like inflorescences of açaí palms comprise both staminate and pistillate flowers [2,16]. Thus, açaí is a monoecious plant species. Açaí berries are spherical and organized into clusters formed by hundreds of individual fruits (Figure 2d). Each açaí berry has a diameter of 1.0 to 2.0 cm and an average mass of 1.5 g [27]. Externally, açaí fruit has a dark purple epicardium (Figure 2e). The maturity of açaí fruit is determined by its outermost color. At peak ripeness, the skin of açaí berries appears black [29]. Internally, the fruit contains a seed (Figure 2d) surrounded by an oleaginous pulp (mesocarp) that is 1.0 to 2.0 mm thick. Both the epicarp and mesocarp are edible and possess a flavor similar to that of a raspberry [27]. Although the açaí seed only weighs between 0.6 and 2.8 g and varies from 0.6 to 2.5 cm in diameter, it represents up to 85% of the volume of an individual açaí berry. Açaí seeds have a fibrous tegument, hard endocarp, and small embryo [2,15,27,29,30].

## 3. Phytochemical Profiles

Notably, the disease-preventing effects of açaí are related to its composition of bioactive phytochemicals. The major constitutive phytochemicals of açaí are present throughout various parts of the plant.

### 3.1. Fruit

**Figure 3:**
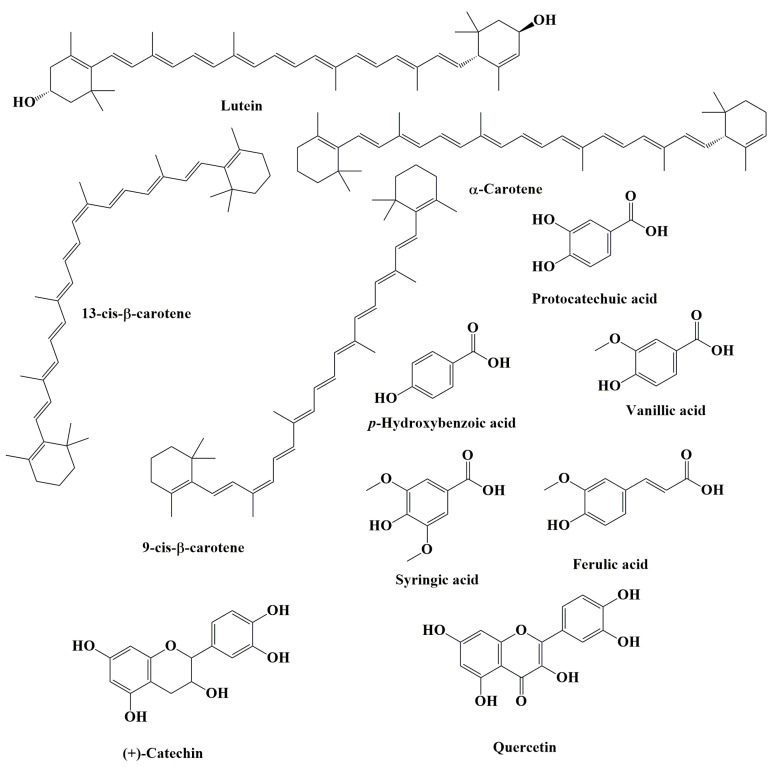
Major phytochemical compounds present in açaí fruit and oil.

In the açaí fruit, the polyphenols are the most significant constituent of the chemical profile. Major secondary polyphenol metabolites include anthocyanins (ACNs) and proanthocyanidins (PACs), in addition to other flavonoids. Several phenolic acids (e.g., ferulic acid, vanillic acid, syringic acid), flavonoids (e.g., catechin and quercetin), lignans, and procyanidin oligomers have been reported in the phytochemical profile of açaí fruit [20,31,32]. The predominant carotenoids (terpenoids) found in açaí fruit are lutein, α-carotene, 13-cis-β-carotene, and 9-cis-β-carotene (Figure 3) [33].

### 3.2. Oil

In commercial settings, açaí pulp is clarified through the extraction of açaí oil via a water-insoluble filter cake. Data has demonstrated the presence of various phenolic acids (e.g., protocatechuic acid, *p*-hydroxybenzoic acid, vanillic acid, syringic acid, ferulic acid) and a flavonoid, (+)-catechin, in açaí oil (Figure 3) [34].

### 3.3. Pulp and Seed

**Figure 4:**
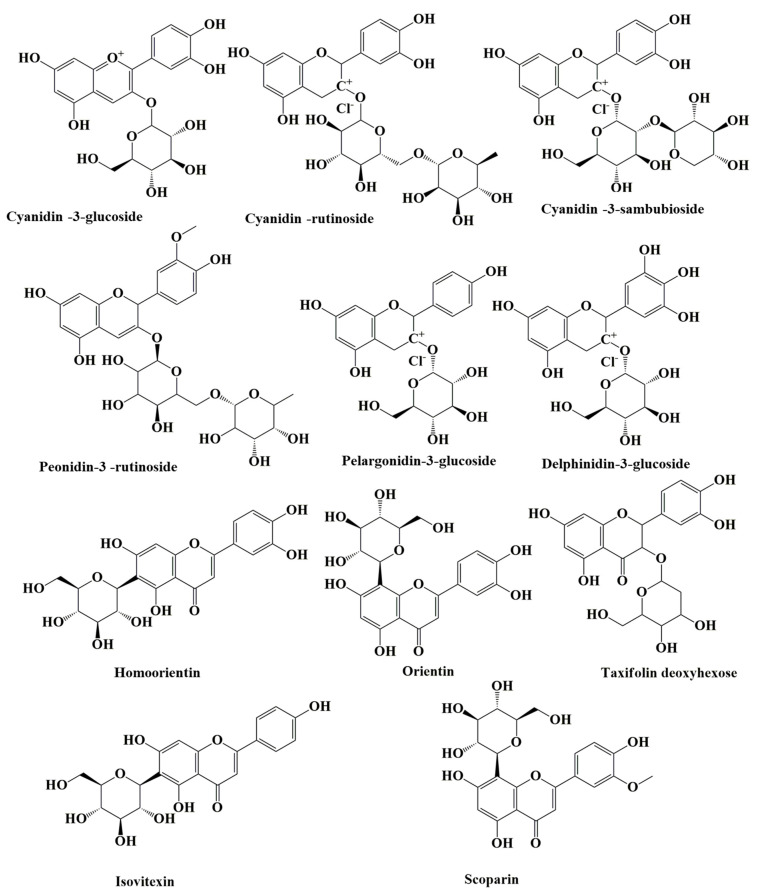
Major phytochemical compounds present in açaí fruit seeds and pulp.

Both the pulp and seed of açaí are rich in phytochemicals. While the chemical profile of açaí seeds consists of 28.3% polyphenols, açaí pulp contains 25.5% polyphenols, the majority of which are cyanidin 3-glucoside and cyanidin 3-rutinoside (Figure 4) [7,35,36,37]. Cyanidin-3-rutinoside has been recorded as the most prevalent anthocyanin in açaí pulp, followed by cyanidin-3-glycoside. Other anthocyanins, such as cyanidin-3-sambubioside, peonidin-3-rutinoside, pelargonidin-3-glucoside, and delphinidin-3-glucoside, have also been found in freeze-dried açaí pulp (Figure 4). Moreover, the presence of other flavonoids, such as homoorientin, orientin, taxifolin deoxyhexose, isovitexin, and scoparin, has been reported in analyses of the composition of freeze-dried açaí pulp (Figure 4) [38].

**Figure 5:**
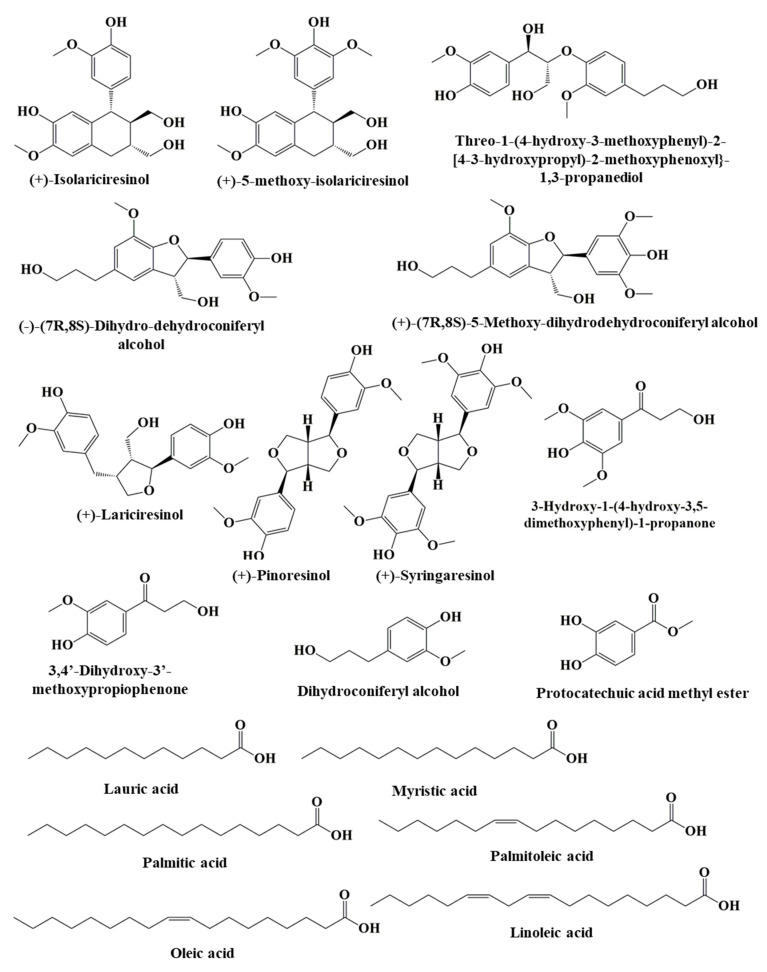
Major lignans and fatty acids present in açaí fruit seeds and pulp.

Of the non-cyanidin constituents of açaí, phenolic acids (non-flavonoids), such as 3,4-dihydroxybenzoic acid; *p*-hydroxybenzoic acid; vanillic acid; caffeic acid; syringic acid; and ferulic acid, have been identified in samples of freeze-dried açaí pulp [39]. It has been noted that freeze-dried açaí pulp contains lignan isolates, such as (+)-isolariciresinol; (+)-5-methoxy-isolariciresinol; erythro-1-(4-hydroxy-3-methoxyphenyl)-2-[4-(3-hydroxypropyl)-2-methoxyphenoxy]-1,3-propanediol; threo-1-(4-hydroxy-3-methoxyphenyl)-2-[4-(3-hydroxypropyl)-2-methoxyphenoxy]-1,3-propanediol; (−)-(7R,8S)-dihydrodehydroconiferyl alcohol; (+)-(7R,8S)-5-methoxy-dihydrodehydroconiferyl alcohol; (+)-lariciresinol; (+)-pinoresinol; (+)-syringaresinol; 3-hydroxy-1-(4-hydroxy-3,5-dimethoxyphenyl)-1-propanone; 3,4′-dihydroxy-3′-methoxypropiophenone; dihydroconiferyl alcohol; and protocatechuic acid methyl ester (Figure 5) [40]. Assays of freeze-dried açaí pulp have also revealed the presence of a variety of saturated fatty acids, monounsaturated fatty acids, polyunsaturated fatty acids, sterols, and amino acids [38]. Similarly, açaí seeds are rich in fatty acids, including lauric, myristic, palmitic, palmitoleic, oleic, and linoleic acids (Figure 5).

### 3.4. Leaf and Root

**Figure 6:**
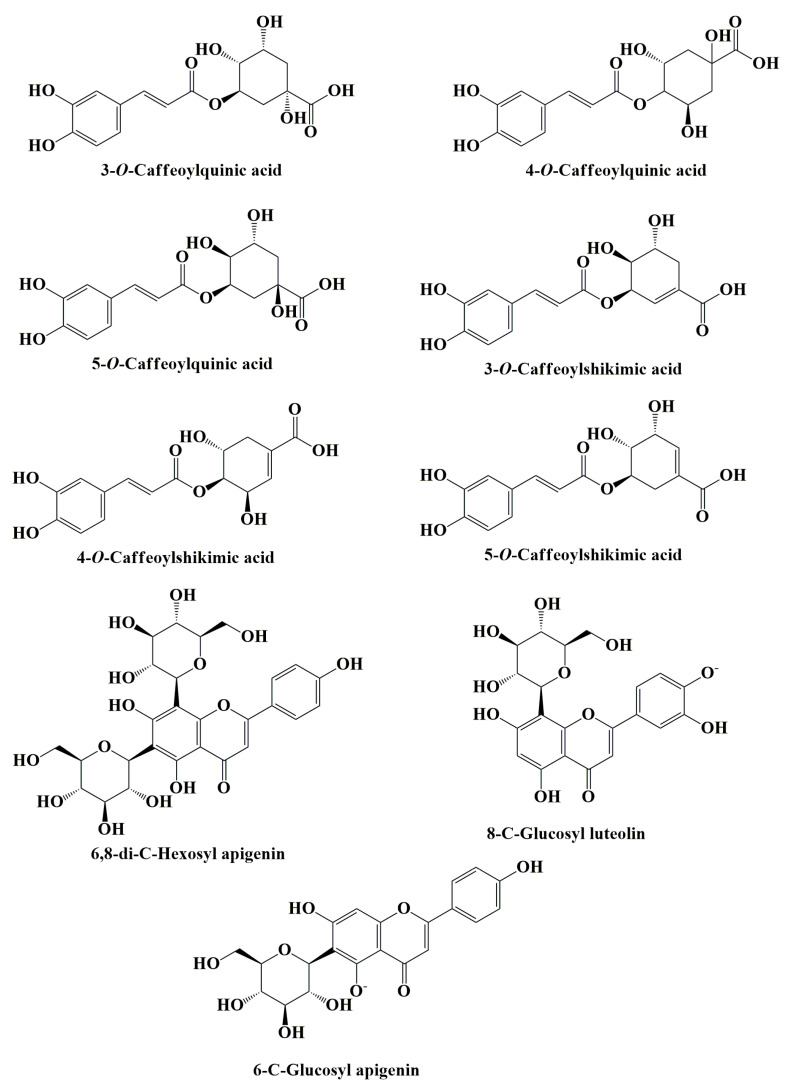
Major phytochemical compounds present in açaí leaves and roots.

Furthermore, it has been demonstrated that açaí leaf and root extract contain several phenolic hydroxycinnamic acid compounds, including 3-*O*-caffeoylquinic acid, 4-*O*-caffeoylquinic acid, and 5-*O*-caffeoylquinic acid [41]. Açaí root, in particular, contains other hydroxycinnamic acids, such as 3-*O*-caffeoylshikimic acid, 4-*O*-caffeoylshikimic acid, and 5-*O*-caffeoylshikimic acid (Figure 6). Additionally, açaí leaf consists of apigenin di-*C*-glycosides (ACGs), a group of flavonoids, including: 6,8-di-*C*-hexosyl apigenin; 6,8-di-*C*-hexosyl apigenin sulfate; 6-*C*-hexosyl-8-*C*-pentosyl apigenin isomers; 6-*C*-glucosyl luteolin, or homoorientin; 6-*C*-pentosyl-8-*C*-hexosyl apigenin isomers; 8-*C*-glucosyl luteolin; and 6-*C*-glucosyl apigenin (Figure 6) [42].

## 4. Biological and Pharmacological Effects

### 4.1. Methodology for Literature Search and Included Studies

**Figure 7:**
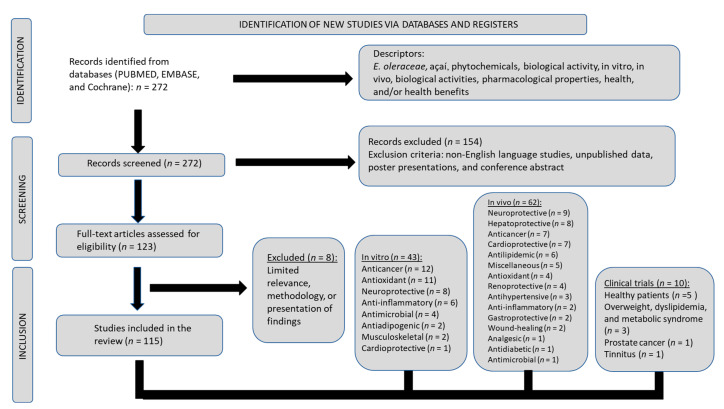
Flow diagram showing the study selection.

This review was designed on the basis of the following focal question: “What are the described health effects of açaí?” A literature search through PubMed, Cochrane, Embase, and Google Scholar databases was conducted to identify studies performed with açaí in relation to its health benefits, implications in disease, or both. Keywords directing the investigation included *E. oleracea*, açaí, phytochemicals, biological activity, in vitro and in vivo biological activities, pharmacological properties, and health benefits. Adherence to the Preferred Reporting Items for Systematic Reviews and Meta-Analysis (PRISMA) guidelines was maintained throughout the process of data collection [43,44]. The literature search and selection process utilized in this study are depicted in Figure 7.

Eligible studies published from 2004 to 2022 were included in this review. Exclusion criteria were non-English language studies, unpublished data, poster presentations, and conference abstracts. For each clinical trial study, the detection, selection, and reporting of bias was utilized to carefully consider and evaluate risk of bias. Patient inclusion, intervention category, outcomes analyses, missing events, and data were also examined. Assessments of both bias and quality were performed in alignment with directives of the Cochrane Handbook for Systematic Reviews of Interventions [45]. The descriptive results of biases that were identified in the included in vivo animal studies followed Systemic Review Center for Laboratory Animal Experimentation (SYRCLE) guidelines [46]. We found 43 in vitro studies, 62 in vivo studies utilizing animal models, and ten clinical trials that aligned with our proposed focal question for this review. The patient populations involved in the clinical trials in this report are noteworthy. In the included studies, the distribution and distinction of patient populations are as follows: five with healthy subjects; one with patients with tinnitus; three with overweight individuals, one of which included patients diagnosed with metabolic syndrome (MetS); and one with patients with prostate cancer.

### 4.2. Preclinical Studies

Numerous preclinical studies have revealed the effects of açaí in vitro (Table 1) and in vivo (Table 2). A risk of bias assessment was performed for each animal study (Supplementary Table S1). The most relevant biological and pharmacological effects of açaí are described below.

#### 4.2.1. Antioxidant Activity

Among the health implications of açaí included in this discussion, antioxidant and anti-inflammatory faculties have been documented most frequently in the current literature. A large quantity of in vitro evidence exists in support of the antioxidant capacity of several compounds (e.g., polyphenols, flavonoids, anthocyanins) present in açaí [41,42,142,143]. Brunschwig et al. [42] evaluated the in vitro antioxidant effect of açaí root and leaflet extracts using ferric reducing antioxidant power (FRAP), oxygen radical absorption capacity (ORAC), and 2,2-diphenyl-1-picrylhydrazyl (DPPH) tests. In this study, both açaí root and leaflet extracts were found to exhibit powerful antioxidant activity against superoxide anion radical and promote the inhibition of liposome, hydroxyl radical, peroxyl radical, and DPPH radical oxidation. Evidence suggests that these effects were induced by hydroxycinnamic acids and ACGs present in açaí root and leaflet, respectively [27,144]. In a study evaluating the antioxidant capacity of several commercial samples of powdered açaí pulp, Carvalho et al. [39] demonstrated that all brands of açaí pulp presented activity in ORAC and 2,2′-azino-bis(3-ethylbenzothiazoline-6-sulfonic acid) (ABTS) assays. The authors reported data supportive of antioxidant effects correlated to the presence of phenolic compounds in açaí.

**Figure 8:**
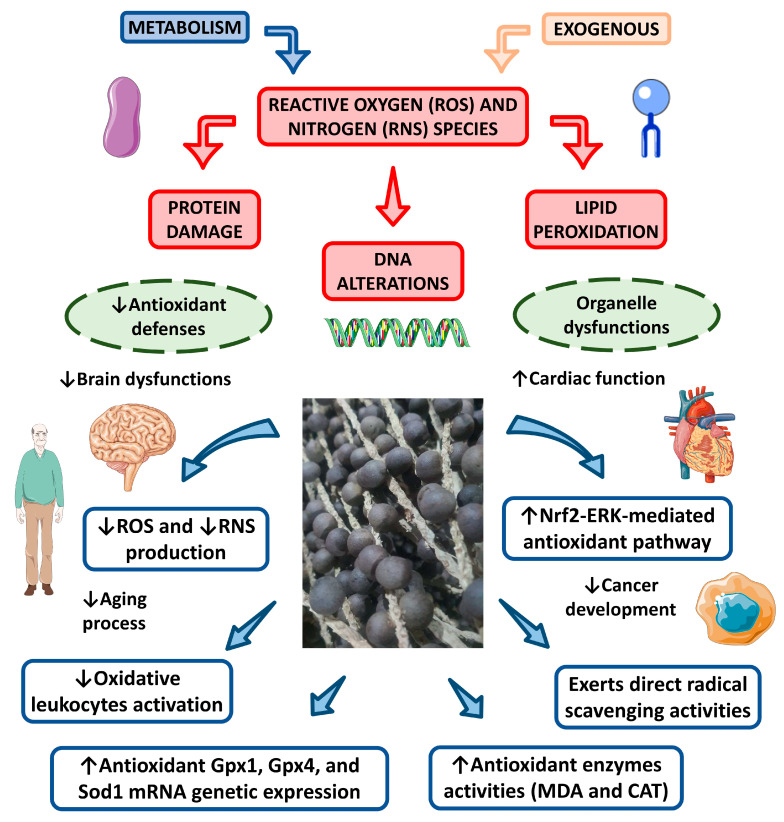
Antioxidant effects of açaí. ROS cause protein damage, DNA alterations, and lipid peroxidation throughout biological systems. Açaí decreases the production of oxidative products and increases cellular antioxidant capacity. Symbols and abbreviations: ↑: increase; ↓: decrease; CAT: catalase; GPx-1: glutathione peroxidase-1; GPx-4: glutathione peroxidase-4; MDA: malonaldehyde; Nrf2-ERK: nuclear transcription factor-erythroid 2-related factor 2-extracellular signal-regulated kinases; ROS: reactive oxygen species; RNS: reactive nitrogen species; SOD1: superoxide dismutase 1.

In a similar manner, Earing et al. [145] evaluated the composition and antioxidant capacity of açaí food supplements in several different formulations (e.g., capsule, powder, frozen pulp, liquid) according to chemical profiles and antioxidant properties. Notably, over half of the açaí supplements were found to either consist of little to no açaí berry or enough water to significantly dilute the chemical constituents of the fruit. Moreover, few supplements contained unlisted ingredients that altered the chemical properties of the açaí products. Nonetheless, a strong positive correlation (r = 0.978) was identified between antioxidant capacity and total phenol content. Further, Costa et al. [75] evaluated the phytochemical profile of hydroethanolic açaí extract from a commercial dietary açaí powder supplement and investigated the in vitro influence of açaí phytochemicals on angiogenesis and oxidative biomarkers in human microvascular endothelial cells (HMEC-1). Analysis of the hydroethanolic açaí extract revealed the presence of considerable quantities of anthocyanins, primarily cyanidin-3-O-rutinoside, and various flavonoids with promising health implications [75]. HMEC-1 treated with hydroethanolic açaí extract demonstrated decreased reactive oxygen species (ROS) production, upregulated antioxidant activity of catalase (CAT) and superoxide dismutase (SOD), and increased antiangiogenic activity without cytotoxicity [75]. Figure 8 summarizes the antioxidant effects of açaí.

#### 4.2.2. Anti-Inflammatory Activity

**Figure 9:**
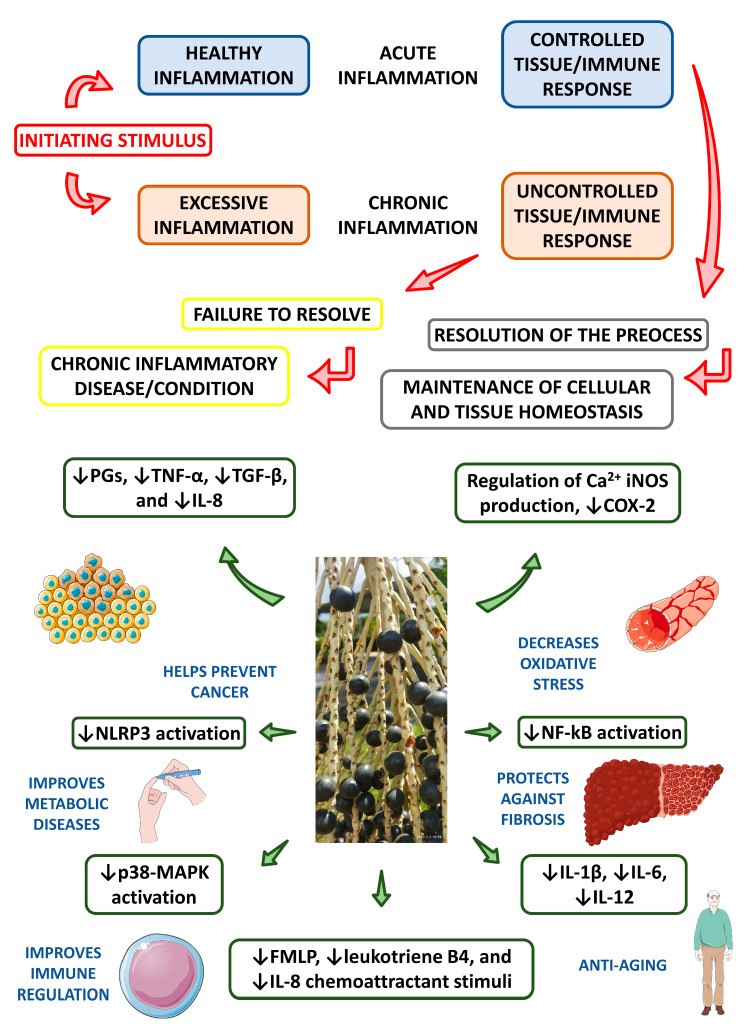
Anti-inflammatory effects of açaí. Açaí improves anti-inflammatory status by directly reducing the synthesis of proinflammatory cytokines and expression of proinflammatory signaling pathways. Symbols and abbreviations: ↑: increase; ↓: decrease; Ca^2+^: calcium; COX-2: cyclooxygenase-2; FMLP: N-formylmethionyl-leucyl-phenylalanine; IL-1β: interleukin-1β; IL-6: interleukin-6; IL-8: interleukin-8; IL-12: interleukin-12; NF-κB: nuclear factor-κB; iNOS: inflammatory nitric oxide synthase; PGs: prostaglandins; MAPK: mitogen-activated protein kinase; TGF-β: transforming growth factor-β; TNF-α: tumor necrosis factor-α.

Injury, toxins, infection, genetic defects, and trauma can induce resident immune cell activation [4]. Subsequent signaling and secretion of chemokines and cytokines, such as cyclooxygenase 2 (COX-2), tumor necrosis factor-α (TNF-α), and nuclear factor-κβ (NF-κβ), recruit immune cells to the affected region and cause inflammatory infiltration [54,56,146,147]. Historically, in vitro models have been used to evaluate the anti-inflammatory effects of açaí. Dias et al. [147] utilized noncytotoxic concentrations of açaí extract in human colon myofibroblast *CCD-18Co* cells to investigate the inflammatory protein expression, ROS suppression, and anti-inflammatory activity of açaí phenolic compounds. Açaí extract was noted to decrease COX-2, TNF-α, and NF-κβ expression induced by lipopolysaccharide (LPS) in human colon myofibroblasts. Because the downregulation of biomarkers, such as COX-2, TNF-α, and NF-κβ, is vital to the reduction of inflammation, these results provide further evidence of anti-inflammatory activities of phenolic compounds in açaí. Another in vitro study performed by Machado et al. [148] demonstrated that hydroalcoholic extract of açaí pulp and bark prevented the increase of proinflammatory cytokines, such as interleukin-1β (IL-1β), interleukin-6 (IL-6), TNF-α, and interferon-γ (IFN-γ), as well as ROS and nitric oxide (NO), on an inflammatory macrophage model. In this study, açaí was also shown to promote the increase of anti-inflammatory interleukin 10 (IL-10) levels. Thus, data suggests the capacity of açaí as an agent of inflammatory regulation and inhibition [56,57,148]. Figure 9 shows the potential anti-inflammatory actions of açaí.

#### 4.2.3. Antinociceptive and Analgesic Activity

Pain can have a negative impact on quality of life, as well as the performance of daily activities. Currently, nonsteroidal anti-inflammatory drugs (NSAIDs) and opioids are common pharmacological options of the treatment of pain. The anti-inflammatory, antihypertensive, antioxidant, and vasodilatory activities of açaí has resulted in the exploration of its value as an antinociceptive and analgesic agent [56,95,97,118,149]. According to Sudo et al. [95], the use of açaí seed extract reduced nociceptive responses to acute/inflammatory pain, including acetic acid-induced writhing, thermal hyperalgesia, and carrageenan-induced thermal hyperalgesia in mice. The antinociceptive responses to açaí were dose-dependent. Furthermore, açaí extract diminished the neurogenic and inflammatory phases resulting from intraplantar injections of formalin and prevented chronic pain, including mechanical allodynia and thermal hyperalgesia, induced by spinal nerve ligation. Açaí displayed noteworthy antinociceptive action through multiple pathways and, therefore, may be considered in the production of new analgesic therapeutics. Additionally, Marinho et al. [150] showed that extracts from açaí flowers and spikes have antinociceptive activity in rat models. The flower extract has significant peripheral activity, reducing the total number of abdominal contortions by up to 50% in an acetic acid-induced abdominal writhing pain model. Although none of the açaí extracts were able to change the analgesia indices in a hot plate pain model, higher dosages of açaí achieved positive spinal antinociceptive effects. Açaí may have potential as therapeutic in the treatment and management of pain.

#### 4.2.4. Antimicrobial Activity

Due to growing antimicrobial resistance, the pharmaceutical field is constantly seeking new alternatives to oppose relevant pathogens. The high polyphenol content of açaí has been associated with antimicrobial activity [60]. One study has investigated the effects of açaí oil (EOO) complex against *Escherichia coli, Pseudomonas aeruginosa*, *Streptomyces aureus*, and *Enterococcus faecalis* [151]. EOO complexes containing β-cyclodextrin (β-CD) or hydroxypropyl-β-cyclodextrin (HP-β-CD) were also investigated. Results showed a modulatory antibacterial response of EOO, EOO-β-CD, and EOO-HP-β-CD and revealed that EOO can successfully form inclusion complexes, especially with β-CD. Minimum inhibitory concentration (MIC) demonstrated that the inclusion complexes in EOO-β-CD and EOO-HP-β-CD exhibited antibacterial effects against Gram-positive and Gram-negative strains and were considerably more potent than pure EOO. Moreover, EOO and most of its complexes exhibited a synergistic effect with ampicillin against *E. coli* [21]. Overall, data elucidating the antimicrobial response elicited through these complexes carries is of great importance to the pharmaceutical industry, as açaí may be considered in the development of new forms of microbicidal drugs.

Dias-Souza et al. [62] studied the antimicrobial effects of methanolic extract of açaí pulp against *S. aureus* and found it to be effective against planktonic cells and biofilms of this microorganism. Furthermore, hydroalcoholic extracts produced from dried pulp, leaves, and seeds of açaí demonstrated significant antimicrobial activity against *Clostridium perfringens*, *S. aureus*, and *P. aeruginosa* [63]. Hence, these studies suggest that the use of açaí extract in formulations of phytotherapeutic substances may be a sustainable option for antimicrobial treatment. Further, Silva et al. [24] evaluated the activity of açaí juice on *Leishmania amazonensis* and *Leishmania infantum* and found a reduction in the number of promastigotes, augmented synthesis of ROS, and the induction of cell death phenotypes in both species. The use of açaí juice for 72 h not only engendered protective effects against *L. amastigotes* and strongly diminished IL-17 levels, but also reduced the number of intracellular amastigotes in macrophages infected with *L. amastigotes* and *L. infantum* [24]. Of note, the authors did not observe cytotoxic effects in murine macrophages treated with açaí juice. Murine macrophages often serve as the initial screening tool for bioactivities of natural products on in vivo or human primary cells. Accordingly, these results revealed the leishmanicidal activity of açaí juice against species responsible for the onset of cutaneous and American visceral leishmaniasis without concomitant cytotoxic consequences for the host cell.

#### 4.2.5. Antiulcer Activity

Gastric ulcers are one of the most common conditions afflicting humans. Current treatments for gastric ulcers include H2-receptor antagonists, M1-receptor blockers, and proton pump inhibitors. However, these drugs can be costly, have health-associated side effects, and result in relapse. For this reason, more efficacious and inexpensive therapeutics for gastric ulcers are in high demand. Cury et al. [96] investigated the in vivo effects of dried açaí fruit extract (DAE) on ethanol-induced gastric ulcers through animal models and noted gastroprotective activity. In this study, 30 to 100 mg/kg doses of DAE resulted in a 48–83% reduction of ulcerated area. Furthermore, DAE demonstrated in vitro radical scavenger capacity through increased levels of glutathione, normalized levels of SOD, increased CAT activity, and reduced levels of TNF-α levels in comparison to the control group. In sum, these findings indicate that açaí extract may both decrease inflammation and facilitate the maintenance of oxidative balance in gastric mucosa. Thus, açaí is a promising natural option for pharmacotherapy aiming to protect the gastric mucosa.

#### 4.2.6. Neuroprotective Activity

There are limited investigations examining the impact of açaí berry on cognitive function or brain health. The experiments available have established that açaí largely confers its neuroprotection through antioxidant and anti-inflammatory mechanisms, restoration of mitochondrial function, and inhibition of toxic protein aggregation [152,153,154,155]. Antioxidants have a clear role in the neutralization of free radicals and, therefore, the protection of cells against oxidative damage caused by free radicals [14]. Oxidative damage has been linked to the development of chronic illnesses and is the common cytopathology of many neurodegenerative diseases (NDDs) [156,157]. Neuronal degradation and the development of NDDs are generally multifactorial processes incited by a genetics, aging, and environmental factors linked to the progression of oxidative stress, chronic neuroinflammation, mitochondrial dysfunction, anomalous protein accumulation in brain tissues, and excitotoxicity [72,148,155]. Data have shown that neurons unequipped for adequate response to oxidative stress undergo apoptotic or necrotic death [69]. Thus, oxidative stress is a primary mechanism responsible for neuronal degradation. In comparison to other organs, the relative lack of antioxidant enzymes, abundance of readily oxidizable substances, and substantial oxygen requirements in the brain render it more susceptible to free radical damage [72]. Hence, materials rich in antioxidants can afford neuroprotective effects against oxidative damage [156].

The anti-inflammatory potential of açaí extract was evaluated via an in vitro microglial model that substantiated its modulation of nucleotide oligomerization domain (NOD)-like receptor pyrin domain-containing 3 (NLRP3) inflammasome proteins and antioxidant pathways, augmentation of anti-inflammatory cytokines, and reduction of pro-inflammatory cytokines [155]. Microglia were exposed to LPS and nigericin, inducers of inflammation, then treated with açaí. Results confirmed the efficaciousness of açaí in neuroinflammatory prophylaxis and, therefore, the potential of the fruit in the prevention and treatment of neuropsychiatric diseases associated with neuroinflammation. De Souza et al. [157] also validated that açaí attenuated stress-induced inflammatory and oxidative signals in BV-2 microglial cells insulted with LPS. This study evaluated the success of açaí as an anti-neuroinflammatory agent. Similarly, freeze-dried hydroalcoholic açaí berry extract was found to reduce cellular proliferation, release of ROS, proinflammatory cytokines, and caspase-1 protein expression [66]. Furthermore, açaí berry extract exhibited the capacity to induce cell cycle arrest, suggesting the role of açaí as an inhibitor and regulator of the inflammatory response. Another study, investigating the antioxidant and neuroprotective actions of hydroethanolic extracts from six açaí genotypes, illustrated the potent scavenging capability of açaí via ABTS, deoxyribose, and glutathione oxidation assays, as well as human neuroblastoma SH-SY5Y cell lines subjected to hydrogen peroxide (H_2_O_2_). Analysis of ABTS and deoxyribose assays revealed no differences in antioxidant activity amongst the various açaí genotypes. All hydroethanolic extracts were found to reduce ROS produced by H_2_O_2_ in SH-SY5Y cells, elucidating their neuroprotective effects [67].

The vulnerability of the brain to oxidative stress, in addition to its energy requirements for neurotransmission, greatly increases the risk of mitochondrial dysfunction in neural tissue [69]. Data supports the correlation between mitochondrial dysfunction and neuronal death in NDDs [155]. Additionally, the current literature has linked the pathophysiology and cellular modifications of neuropsychiatric illnesses, such as bipolar disorder (BD) and schizophrenia (SCZ), to mitochondrial dysfunction and oxidative stress, respectively [67]. Machado et al. [67] examined the in vitro role of açaí in modulation of mitochondrial function and oxidative metabolism. In this study, freeze-dried hydroalcoholic açaí extract reversed mitochondrial dysfunction incited by rotenone treatment in human neuroblastoma SH-SY5Y cells. Açaí extract not only augmented protein amount and enzyme activity of mitochondrial complex I, but also to reduced cellular ROS and lipid peroxidation [67,72]. Mitochondrial complex I is the first enzyme of the electron transport chain (ETC), a series of protein complexes responsible for oxidative phosphorylation in mitochondria. Restoration of mitochondrial function was primarily achieved through overexpression of NDUFS7 and NDUFS8 nuclear mitochondrial complex I subunit genes and improvement of their proteomic expression [67]. Collectively, these results led the authors to suggest that the neuropharmacological faculty of açaí may warrant its candidacy in the development of drugs used to treat BD, SCZ, and other neuropsychiatric diseases.

Neurodegeneration in the setting of NDDs can cause significantly altered neuron viability. This can lead to the overproduction, inadequate clearance, and ultimately, aggregation of toxic proteins in or around brain tissue [69]. The death of neurons is precipitated by toxic protein accumulation often caused by dysfunctional autophagy [69]. Therefore, normal cellular autophagy is a crucial determinant of neuron viability protein homeostasis in the brain. Wong et al. [70] showed that açaí berry extract significantly improved neuronal cell viability after exposure to beta-amyloid (Aβ), specifically Aβ1-42. In comparison to pure phenolics, the phenolic compounds of açaí extract exhibited more potent effects on Aβ1-42 fibril inhibition and morphological modification. Toxic protein misfolding and accumulation are pathological hallmarks of many NDDs [69]. The ability of açaí extract to efficaciously impede Aβ1-42 aggregation reinforced existing evidence of the neuroprotective effect of this fruit. Furthermore, it can be seen that the molecular mechanisms of açaí berry may contribute to the improvement of protein homeostasis in the brain [69].

Brain injury from seizures can result in neuronal cell death [65]. Factors contributing to seizure-induced neuronal cell death include oxidative stress, mitochondrial dysfunction, altered levels of cytokines, and genetics. Based on this association, Souza et al. [23] examined the possible anticonvulsant and neuroprotective activities of commercial clarified açaí juice and showed that the juice did not modify spontaneous locomotor activity in mice. In this study, four doses (10 µL/g) of the juice provided adequate anticonvulsant activity to augment latencies to both first myoclonic jerk and first generalized tonic–clonic seizure. The administration of açaí significantly reduced the total duration of tonic–clonic seizures induced by pentylenetetrazol. Moreover, açaí juice prevented electrocortical modification, as well as lipid peroxidation, caused by the use of pentylenetetrazol in the cerebral cortex [23]. Importantly, this is the first data to demonstrate the neuroprotective effects of açaí against seizures and seizure-related oxidative stress.

Many neurodegenerative diseases eventually lead to symptoms of depression. Because depression has been linked to oxidative stress, the antidepressant effects of açaí berry have also been explored [156]. Açaí juice has been shown to exhibit antidepressant actions similar to those of imipramine, which inhibits neuronal reuptake of norepinephrine and serotonin neurotransmitters. Currently, the similitude between these two substances and their antiaging and antidepressant effects is thought to be due to their roles in the prevention of lipid peroxidation and increase of telomerase reverse transcriptase mRNA expression [72,156,157,158]. Specifically, açaí juice provides protection against neuronal loss related to the depressive-like state and increased nitrite levels in hippocampal cells [104]. Additionally, the reduction of oxidative damage in the brain as a result of açaí treatment may be a productive intervention of the aging process [84]. Sun et al. [156] investigated the effects of açaí pulp supplementation in *Drosophila melanogaster* fed a high-fat diet. This study found that açaí supplementation increased the transcription of genes related to a small heat-shock-related protein and two detoxification genes. Açaí also reduced transcription of phosphoenolpyruvate carboxykinase (PEPCK), a critical gene in the appropriate regulation of gluconeogenesis. Moreover, açaí increased the lifespan of females subjected to oxidative stress, suggesting that açaí improves survival [159]. Açaí may be an effective antagonist of the deleterious effects of high-fat diets and oxidative stress, which accelerate the aging process [87,115,116,117,158,159].

#### 4.2.7. Antilipidemic Activity

Dyslipidemia is precipitated by disruptions in lipid metabolism that result in chronically elevated serum lipids and, therefore, increased predisposition to CVDs, obesity, atherogenic processes, diabetes, and metabolic syndrome (MetS) [106,160]. Dietary unsaturated fats can reduce the risk of CVD. Several studies have shown that the consumption of açaí oil and its rich content of unsaturated fatty acids may benefit lipid profiles [18]. Liz et al. [159] investigated the effects of the daily consumption of 200 mL of açaí juice in adults and discovered increased levels of high-density lipoprotein cholesterol (HDL) in comparison to baseline levels. Moreover, Bem et al. [113] demonstrated that açaí seed extract, in conjunction with exercise training, reduced total cholesterol levels by 81.2% in diabetic rats. In addition, Faria et al. [105] examined the actions of açaí oil on hyperlipidemia induced by *Cocos nucifera* L. saturated fat in rats. Although no alterations in triglycerides were noted, there were reductions in total cholesterol and low-density lipoprotein cholesterol (LDL). A treatment regimen of both açaí oil and simvastatin, a common anticholesterolemic drug, also prevented the formation of atheromatous plaques in the vascular endothelium of rats [106,109]. Comparably, a study performed by Souza et al. [106] evaluated the effects of açaí oil in Triton-induced dyslipidemia in rats. The results showed that animals treated with açaí oil and simvastatin exhibited significantly reduced total cholesterol, LDL, and triglyceride levels, as well as increased HDL levels. Thus, the authors concluded that açaí oil is antihypertriglyceridemic, anticholesterolemic, and advantageous in the treatment of dyslipidemia [107]. For this reason, açaí oil may also have potential as a preventive agent for CVDs [20,120].

**Figure 10:**
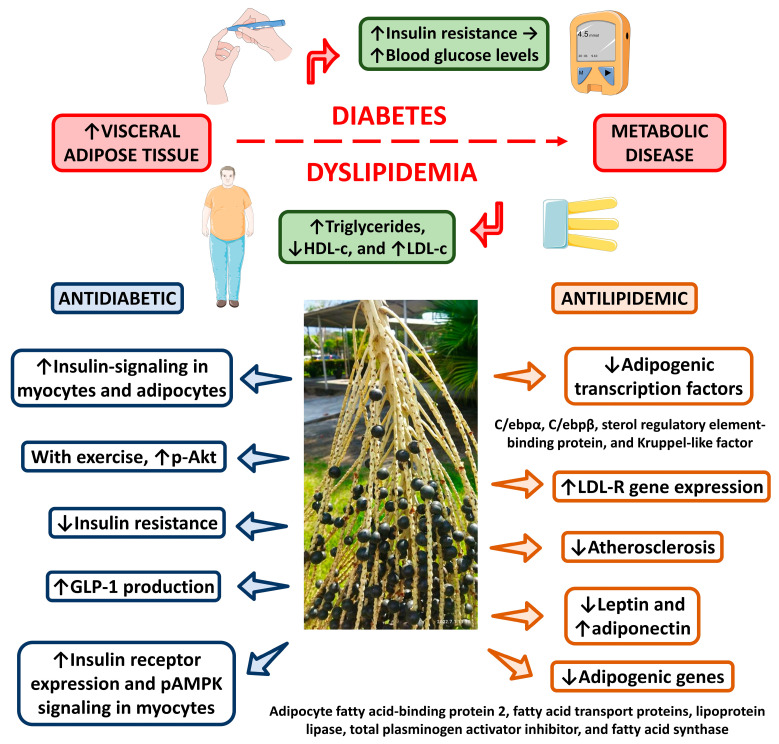
Antidiabetic and antilipidemic effects of açaí. Açaí improves glycemic control and exerts antilipidemic effects via various mechanisms. Symbols and abbreviations: ↑, increase; ↓, decrease; GLP-1, glucagon-like peptide 1; HDL, high-density lipoprotein cholesterol; LDL, low-density lipoprotein cholesterol; LDL-R, low-density lipoprotein cholesterol receptor; Akt, protein kinase B; AMPK, 5′ adenosine monophosphate-activated protein kinase.

Souza et al. [107] proposed that the antilipidemic effects promoted by açaí primarily occur via increased expression of genes involved in cholesterol secretion and biosynthesis, including ATP-binding cassette, subfamily G transporters (ABCG5 and ABCG8), and LDL receptor (LDL-R) genes. This study evaluated the effects of açaí pulp in rats with diet-induced hypercholesterolemia. The experimental group fed a standard diet supplemented with açaí displayed a significant reduction in total cholesterol and LDL levels and an increase in HDL levels. Additionally, there was an increase in fecal cholesterol excretion and enhanced expression of the LDL-R, 7α-hydroxylase, subfamily G transporters, and ATP-binding cassette genes in the rats [115]. These findings suggest that the consumption of açaí pulp positively effects diet-induced hypercholesterolemia by augmenting the expression of ATP-binding cassette, subfamily G transporters, and LDL-R genes [115,160,161,162]. Figure 10 shows an overview of antilipidemic effects of açaí.

#### 4.2.8. Hepatoprotective Activity

Globally, the most prevalent liver condition is nonalcoholic fatty liver disease (NAFLD) [111]. NAFLD is an umbrella term for a variety of progressive illnesses, including steatosis, steatohepatitis, cirrhosis, and hepatocellular carcinoma. Despite the multifactorial onset and progression of NAFLD, specific intracellular contributory factors, such as inflammation, oxidative stress, mitochondrial dysfunction, altered endoplasmic reticulum (ER) homeostasis, and apoptosis, have been identified [115]. Therefore, the phenolic compounds of açaí are regarded as prospective therapeutic agents in the treatment of NAFLD due to their high antioxidant and anti-inflammatory capacities [47,163,164,165]. Freitas et al. [115] tested the actions of an aqueous açaí extract in HepG2 cells and its effects on inflammation, oxidative stress, and ER stress. These activities were also tested in a murine model of diet-induced NAFLD. Notably, açaí exhibited potent in vitro antioxidant capacity. In vivo açaí extract administration (3 g/kg) attenuated liver damage, as evidenced by decreased levels of alanine aminotransferase (ALT) and serum TNF-α, a reduced number of inflammatory cells, and decreased lipid peroxidation and carbonylation of proteins [163]. Fundamentally, the results of this study propound the idea that açaí extract may have hepatoprotective activity and, thus, the capacity to prevent the progression of liver damage associated with NAFLD [111,115,163]. Additionally, Song et al. [163] evaluated the properties of an anthocyanin-rich extract of açaí fruit in male SPF C57BL/6J mice fed a low-fat diet, high-fat diet, or high-fat diet supplemented with açaí extract for fourteen weeks. Consequently, it was demonstrated that animals treated with açaí presented less hepatic steatosis, obesity, and insulin resistance [35,110,112]. Further, Barbosa et al. [110] showed that the use of pasteurized frozen açaí pulp in female rats fed a high-fat diet two weeks before mating, as well as during gestation and lactation, could improve liver steatosis and reduce liver weight, serum cholesterol, and hepatic fat content. In the offspring, the high-fat diet supplemented with açaí also reduced liver weight and serum cholesterol, suggesting that açaí supplementation may attenuate NAFLD and protect offspring from the detrimental effects of excess lipids in the setting of a high-fat maternal diet [108].

Another study revealed that the use of açaí seed flour could prevent obesity-induced hepatic steatosis in mice [108]. In this study, dietary incorporation of 15% or 30% açaí seed flour led to improved lipid profiles and protective effects against weight gain. The açaí-supplemented diet also reduced lipogenesis, which contributed to the inhibition of NAFLD development. Moreover, açaí consumption was found to influence the modulation of proteins involved in cholesterol excretion and production [35,112]. Increased fecal excretion of bile acids and cholesterol was observed in the setting of a diet supplemented with açaí. Comprehensively, this study evinces the use of açaí seed flour as a tool for the prevention of obesity development and its complications, especially hepatic steatosis. In addition, a study performed by Bem et al. [113] evaluated the use of açaí seed extract and exercise training on hepatic steatosis in diabetic rats and discovered that the açaí treatment, in combination with exercise, could reduce total cholesterol (81.2%), aspartate aminotransferase (AST) (51.7%), hepatic triglycerides, (66.8%), and steatosis (72%) compared to a sedentary control group. Açaí supplementation, in conjunction with exercise training, decreased the expression of hepatic lipogenic proteins and augmented both antioxidant defense and cholesterol transporters in animals with type 2 diabetes mellitus (T2DM). Thus, açaí treatment, combined with exercise, may provide protection against both hepatic steatosis and oxidative stress [105,117,118,138].

The effects of filtered açaí pulp on the expression of paraoxonase (PON) activity in rats with NAFLD have also been investigated [166,167]. Rats fed a high-fat diet supplemented with açaí displayed increased hepatic and serum PON-1 activity, decreased LDL oxidation, and upregulated expression of PON1 and APOA1, which encodes apolipoprotein A-I (ApoA-I), in the liver. Overall, the consumption of açaí pulp reduced liver damage, fat infiltration, and triglyceride content, suggesting its potential efficacy against hepatic steatosis and liver injuries [114]. A study conducted by Oliveira et al. [35] also indicated açaí as a therapeutic agent in the prevention of liver steatosis. In this report, the use of açaí seed extract reduced cholesterol accumulation and triglycerides in the liver and, therefore, reduced hepatic steatosis. The increased expression of lipogenic proteins such as sterol regulatory element binding protein-1c (SREBP-1c) and hydroxymethylglutaryl-coenzyme A reductase (HMG-CoA reductase), as well as the decreased the expression of phosphorylated 5′ adenosine monophosphate-activated protein kinase (p-AMPK), were antagonized by açaí seed extract. In addition, açaí increased the expression of cholesterol excretion transporters [35,115]. The antioxidant effect of açaí seed extract in the liver was illustrated by the restoration of SOD, CAT, and glutathione peroxidase (GPx) activities [35,114]. These findings reinforce the prospective use of açaí as a method to amplify defense mechanisms against oxidative stress through dietary antioxidants.

Alcoholic liver disease (ALD) is the most prevalent cause of advanced liver disease and has been linked to high levels of global mortality and morphological consequences, including alcoholic hepatitis, steatosis, fibrosis, cirrhosis, and hepatocellular carcinoma [114]. The result of excessive ethanol metabolism in the setting of chronic alcohol abuse is substantial ROS and acetaldehyde production. Because metabolite-induced inflammatory factors and oxidative stress are implicated in the progression of ALD, antioxidants capable of reducing ethanol-induced oxidative stress may be considered as preventive agents [114]. Zhou et al. [112] observed that the use of açaí puree in alcohol-treated Wistar rats significantly decreased hepatic enzymes (alkaline phosphatase (ALP), ALT, and ASP), triglycerides, cholesterol, and hepatic index. Moreover, açaí intake attenuated alcohol-induced oxidative stress, as marked by the reduction of malondialdehyde and triglycerides, higher SOD activity, and increased levels of glutathione. There was also a reduction in the hepatic expression levels of inflammatory mediators stimulated by ethanol metabolic processes, such as IL-8, TNF-α, NF-κB, TGF-β, and CD-68. Additionally, treatment with açaí attenuated histopathological liver damage, including severe steatosis and inflammatory cell infiltration [114]. The anti-inflammatory and antioxidative activities of açaí may warrant its application in the treatment of ALD.

#### 4.2.9. Antidiabetic Activity

Worldwide, T2DM is a serious public health crisis reaching epidemic proportions [117]. Diabetes is a major cause of kidney failure, cardiovascular events, blindness, and lower limb amputation. T2DM is associated with an increased risk of morphological and metabolic modifications in vital organs such as the liver [105]. In a study conducted with obese mice on a high-fat diet, Silva et al. [108] revealed that the incorporation of 15% or 30% dietary açaí seed flour procured beneficial effects against insulin resistance. Furthermore, after 12 weeks of açaí intake, the animals presented lower serum glucose, insulin, and leptin concentrations. The reduction of lipogenesis induced by açaí seed flour consumption also prevented the development of hypertrophic obesity [112]. Bem et al. [113] demonstrated that the use of the açaí seed extract and exercise training in diabetic rats can reduce blood glucose by 70.2%. Oliveira et al. [35] examined the impact of açaí seed extract on adiposity and hepatic steatosis and showed its potential to reduce glucose levels in mice treated with a high-fat diet. Hence, açaí has protective activities against obesity and its comorbidities, including insulin resistance and elevated glucose levels. Figure 10 shows the antidiabetic and antilipidemic effects of açaí.

#### 4.2.10. Antihypertensive Activity

Cardiovascular risk factors such as hypertension, dyslipidemia, obesity, diabetes mellitus, and MetS promote endothelial injury due to oxidative stress [120]. This endothelial dysfunction causes an imbalance in vasoconstriction and vasodilation, as well as increased proinflammatory factors and ROS. Oxidative stress, inflammation, and the renin-angiotensin system (RAS) contribute to the development of hypertension [118]. The use of açaí seed extract can exhibit antihypertensive effects in mice fed a high-fat diet, as shown by Santos et al. [164,165,166]. In this study, animals were treated with açaí seed extract (300 mg/kg per day) and enalapril, a common antihypertensive drug. Açaí treatment increased insulin receptor expression, prevented dyslipidemia, and decreased renin and angiotensin II type 1 receptor (AT1 receptor) expression, the latter of which was linked to reduced renin and angiotensin II plasma levels. RAS downregulation in adipose tissue, related to the observed reduction in inflammation and oxidative stress, can contribute to the prevention of hypertension and obesity-related disorders [118]. Moreover, Cordeiro et al. [125] found that açaí seed extract can improve vascular dysfunction and oxidative stress related to hypertension in spontaneously hypertensive animals. The authors observed upregulation of endothelial nitric oxide synthase (eNOS) and SOD1.

ROS play an intricate role in oxidative stress and accompanying myocardial injury. Increased quantities of ROS during heart ischemia can elicit cell membrane destruction, lipid peroxidation, and dysfunction in the antioxidative defense system. For this reason, açaí has been studied as a prospective vasodilatory, anti-inflammatory, and antifibrotic agent [118]. It has been discerned that the consumption of hydroalcoholic extracts of açaí seeds can improve arterial blood pressure in rats submitted to occlusion of the left anterior descendent coronary artery (LAD) by surgery. In this study, açaí treatment prevented the progression of vascular dysfunction, cardiac hypertrophy, fibrosis, and exercise intolerance in rats with previous myocardial injury [118]. The cardioprotective and antihypertensive properties of açaí seed extract might be connected to its anti-inflammatory, antioxidant, and vasodilatory activities [91,95]. Another study performed on rats with a two-kidney, one-clip (2K-1C) renovascular hypertension model verified that the use of açaí seed extract produced antihypertensive effects and prevented endothelial dysfunction and vascular structural changes in 2K-1C hypertension. These results were attributed to interference with NOS activation and oxidative processes, as well as the inhibition of metalloproteinase-2 (MMP-2) activation [118]. Of note, the use of açaí seed extract also can produce endothelium-dependent vasodilation. Rocha et al. [95] showed that the vasodilator action of the extract is dependent on the activation of the nitric-oxide–cGMP pathway and can be involved in endothelium-derived hyperpolarizing factor release. In sum, açaí improves blood pressure through many different pathways, such as vasodilation, increased eNOS activity, and augmented production of arterial elastic fibers [95,118,149].

#### 4.2.11. Cardioprotective Effects

CVDs are currently the leading cause of morbidity and mortality among adults. Although scientific progress has identified a spectrum of different risk factors for cardiovascular pathologies, the current state of prevention, and even treatment, of CVDs is suboptimal [167,168,169]. Over the last few decades, the number of deaths and disability from CVDs has continued to increase, indicating the demand for alternative treatment options for the management and prevention of CVDs. For this reason, açaí has been investigated as a prospective cardiovascular therapeutic agent due to its various cardioprotective bioactive compounds.

Figueiredo et al. [6] treated male Wistar rats with açaí pulp rich in gallic acid and total anthocyanins to evaluate the effects of açaí on animal models of cardiac remodeling. The results revealed that supplementation with açaí pulp significantly attenuated cardiac remodeling after myocardial infarction. Studies conducted by Vilhena et al. [120] and Lavorato et al. [121] described similar findings. However, restoration of normal cardiac function in remodeled mice hearts, as well as improvements in endothelial and kidney functions, inflammation, and oxidative stimuli, were also observed subsequent to açaí treatment.

**Figure 11:**
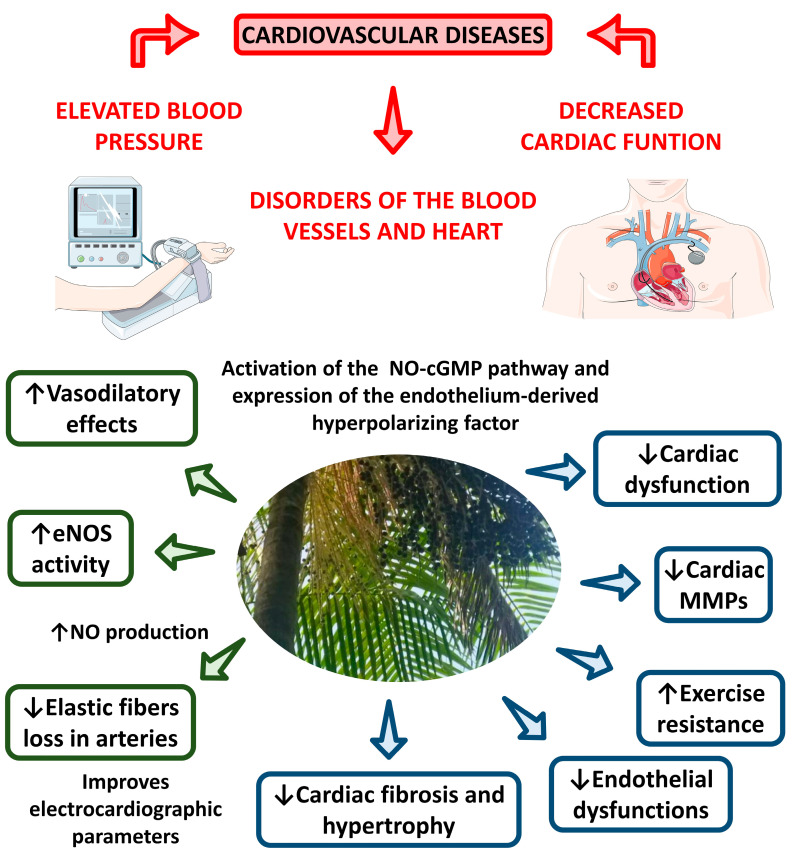
Potential actions of açaí against CVD and cardiovascular risk factors. Açaí improves cardiac function and decreases cardiac fibrosis, exerts vasodilatory effects, diminishes endothelial dysfunction, and ameliorates exercise resistance. Symbols and abbreviations: ↑: increase; ↓: decrease; eNOS: endothelial nitric oxide synthase; MMP: matrix metalloproteinase; NO: nitric oxide; NO–cGMP: nitric oxide cyclic guanosine monophosphate.

Pontes et al. [122] demonstrated that the treatment of male Wistar rats with intravenous aqueous açaí pulp extract intravenously resulted in acutely elevated levels of blood flow. Mathias et al. [123] also found that supplementation with açaí pulp reduced left ventricular dysfunction, oxidative stress, changes in the myocardium metabolism, and metalloproteinases activation in rat models of cardiac remodeling. Similarly, Zapta-Sudo et al. [124] revealed that the use of hydroalcoholic açaí seed extract prevented the development of exercise intolerance, cardiac fibrosis, cardiac dysfunction, and cardiac hypertrophy in male Wistar rats. Cordeiro et al. [125] showed that açaí seed extract could also prevent other vascular dysfunctions, such as vascular remodeling, decreased elastic fibers, altered media/lumen ratio, hypertension, and oxidative damage. Figure 11 illustrates the potential cardioprotective effects of açaí.

#### 4.2.12. Renoprotective Effects

Chronic kidney disease (CKD) is a general term for a group of heterogenous disorders that affect kidney structure and function. Over time, considerable research has been conducted to properly define, classify, and treat CKD. Ultimately, data has indicated that prevention is the most effective way to avoid the development and progression of renal disorders [169,170]. Because many patients with CKD present with pathology related to effects of oxidative stress and inflammation, açaí has emerged as a possible nutritional therapeutic strategy for the prevention and management of CKD.

da Costa et al. [126] found that supplementing the diets of male Wistar rats with hydroalcoholic lyophilized açaí seed extract significantly reduced renal injury and prevented renal dysfunction. da Silva et al. [127] noted that açaí seed extract decreased renal injury, prevented renal dysfunction, and thus exerted renoprotective effects, in a rat model of kidney disease via anti-inflammatory and antioxidative actions. A study conducted by Unis [128] described similar results. Moreover, El Morsy et al. [129] showed that the treatment of male Wistar albino rats with aqueous açaí fruit extract caused dose-dependent attenuation of bilateral renal ischemia/reperfusion-induced renal injury.

#### 4.2.13. Antineoplastic Activity

Over time, the global frequency of cancer, a group of diseases characterized by abnormal cell growth, has continued to increase. Therefore, cancer prevention has become a topic of paramount importance within the scientific field. The chemopreventive and anticarcinogenic potency of açaí has been linked to its ability to decrease the viability of cancer cells, as well as reduce the incidence of tumors and tumor cell proliferation [76,130,153,171,172,173]. In a study performed by Silva et al. [80], the antitumor effects of hydroalcoholic extracts from açaí bark, seed, and fruit was evaluated in vitro using cell lines derived from colorectal and breast adenocarcinomas (human Caco-2 and HT-29 colon adenocarcinoma cells and human MDA-MB-468 and MCF-7 mammary adenocarcinoma cells, respectfully). Results showed that the three extracts from various parts of the açaí plant significantly decreased cancer cell viability by increasing the presence and function of autophagic vacuoles. It was noted that all açaí extracts possessed significant polyphenol content. In another in vitro study, Silva et al. [172] assessed the cytotoxic effects of the extracts of açaí seed, pulp, and fruit in MCF-7 breast cancer cell lines. Açaí seed extract not only reduced the viability of cancer cells via ROS production, but also demonstrated cytotoxic effects and prevented the formation of new cancerous colonies. Moreover, Martinez et al. [174] studied A549 lung carcinoma cell lines treated with açaí seed extract and ascertained that the extract incited cell cycle arrest and increased apoptosis among cancer cells. Although the study lacked a comprehensive evaluation of proapoptotic pathways, the use of açaí extract was found to increase the percentage of cells in G0/G1 cycle phases and contributed to higher numbers of apoptotic cells in comparison to the untreated cells. These results revealed the potent antioxidant activity of açaí seed extract and its protective effects against cancer.

Sibuyi et al. [77] investigated the anticancer properties of gold nanoparticles (AuNPs) synthesis through the use of açaí berry on prostate (PC-3) and pancreatic (Panc-1) cancer cells. The cells were treated with açaí berry extract at concentrations of 50–200 mg/mL, which demonstrated dose-dependent cytotoxicity on the PC-3 cells. Concentrations of açaí berry extract dissolved in various solvents, starting at 50 mg/mL for those of distilled water and those of 10% cyclohexane and at 200 mg/mL those of 50% chloroform, decreased PC-3 cell viability. Açaí berry extract dissolved in 50% ethanol, no matter the concentration, did not produce any effect on PC-3 cells.

Furthermore, Monge-Fuentes et al. [78] studied murine B16F10 melanoma cell lines and found that kinetically stable açaí oil could effectively cause death of 85% of existing melanoma cells via apoptosis. Freitas et al. [79] evaluated the anticancer effects of açaí seed hexane, chloroform, and ethyl acetate extract fractions and discovered that the ethyl acetate fraction was the most effective agent in the reduction of MCF-7 breast adenocarcinoma-derived cell viability through the mechanism of necroptosis. Dias et al. [81] studied malignant colon cancer HT-29 and SW-480 cells treated with polyphenolic açaí juice. Subsequent analyses revealed the ability of açaí to inhibit the growth of malignant cells without causing cytotoxic effects against the normal cells. In a similar fashion, Pacheco-Palencia et al. [82] demonstrated the effects of anthocyanin fractions in açaí fruit in the inhibition of colon HT-29 cancer cell proliferation. Choi et al. [132] demonstrated that dietary supplementation with açaí pulp provides protection against azoxymethane/dextran sulfate sodium-induced colorectal cancer in male ICR mice. Additionally, Hogan et al. [83] treated rat C-6 brain glioma cells and human MDA-468 breast cancer cells with an anthocyanin-rich extract from açaí. Although açaí extract greatly inhibited the proliferation of rat C-6 brain glioma cells, human MDA-468 breast cancer cells did not exhibit a significant response to açaí treatment. Nonetheless, Del Pozo-Insfran et al. [85] treated human HL-60 leukemia cells with açaí pulp extracts and found that the polyphenolic fractions present within the extracts played a crucial role in the reduction of leukemia cell proliferation from 56 to 86% and also increased cancer cell apoptosis.

Fragoso et al. [76] evaluated the antitumor effect of supplementation with the lyophilized pulp of açaí using a model of carcinogenesis associated with colitis in male Wistar rats. This study concluded that lyophilized açaí pulp decreased the proliferation of tumor cells and incidence of tumors with high-grade dysplasia and increased the gene expression of negative regulators of cell proliferation. Upon inspection of the açaí pulp constituents, researchers identified the presence of anthocyanins (e.g., cyanidin 3-*O*-glucoside, cyanidin 3-rutinoside) and carotenoids, especially β-carotene and lutein. Hence, lyophilized açaí pulp was found to have the potential to exert antitumor activity. Additionally, Fragoso et al. [131] demonstrated the anticancer effects of açaí pulp powder through its inhibition of chemically induced carcinogenesis in the colons of Wistar rats. Similarly, Romualdo et al. [133] studied the implications of spray-dried açaí pulp in chemically induced mouse colon carcinogenesis and found that it displayed antineoplastic actions. Monge-Fuentes et al. [78] studied C57BL/6 female mice and assessed the effects of kinetically stable açaí oil nanoemulsion against melanoma tumors, discovering that açaí significantly decreased their volumes. In their study on urinary bladder cells in male Swiss mice, Fragoso et al. [175] found that açaí fruit intake could prevent carcinogenesis via the reduction of DNA damage, tumor cell proliferation, and p63 expression. Comparatively, Lee et al. [176] concluded that açaí berry extract did not effectively prohibit gastric carcinogenesis induced by chronic infections of *Helicobacter felis* in mice.

**Figure 12:**
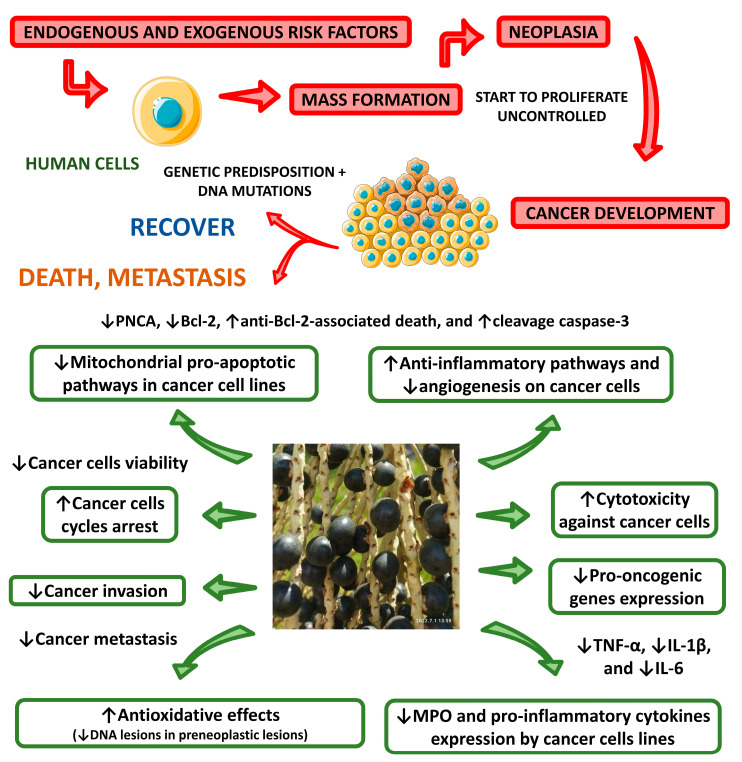
Potential anticancer effects of açaí. Açaí protects against neoplasia through cancer cell cycle arrest, direct cytotoxicity, and reduction of oncogenic gene expression. Due to these actions, açaí reduces cancer cell invasion, augments cancer cell apoptosis, and diminishes cancer cell angiogenesis. Symbols and abbreviations: ↑: increase; ↓: decrease; Bcl-2: B-cell lymphoma 2; IL-1β: interleukin-1β; IL-6: interleukin-6; PNCA: proliferating cell nuclear antigen; TNF-α: tumor necrosis factor-α.

Stoner et al. [134] investigated the effects of dietary açaí flakes (5%) in rats treated with the carcinogen N-nitrosomethylbenzylamine for 5 weeks. Açaí treatment was found to induce augmented serum antioxidant capacity through the reduction of levels of IL-5 and GRO/KC (the rat homologue for human IL-8) in the blood. The authors concluded that the proanthocyanidins and anthocyanins were responsible, at least in part, for the chemopreventive activity of açaí. Perini et al. [26] discussed the anticancer potential of various açaí extracts. These authors determined that the anticancer effects of açaí could be described by a variety of anti-inflammatory, antiproliferative, and proapoptotic effects. The anti-inflammatory actions of açaí protected animal models of cancer against tumor progression and cancer-related inflammatory consequences via the upregulation of IFN-γ and the downregulation of many proinflammatory cytokines and enzymes, such as IL-5, IL-8, TNF-α, IL-1β, myeloperoxidase (MPO), and IL-6. The antiproliferative actions of açaí, such as the downregulation of proliferating cell nuclear antigen (PCNA), p63, and Ki-67, have been shown to decrease the persistence, proliferation, and metastasis of different cancerous tumors. The proapoptotic effects of açaí berry extract, including the upregulation of caspase-3 and the downregulation of Bcl-2, may significantly decrease cancer cell viability and, therefore, tumor progression. Overall, many authors agree that the anticancer potential of açaí is derived mainly from its polyphenolic contents, especially anthocyanins [177]. These anthocyanins are warranted for use as potent antiangiogenic bioactive compounds against angiogenesis-dependent diseases, such as cancer. Figure 12 shows various cellular and molecular mechanisms of the anticancer effects of açaí.

### 4.3. Clinical Studies on Açaí and Human Health

In addition to preclinical studies, numerous clinical trials have evaluated the effect of açaí on different aspects of human health. These studies are summarized in Table 3. The descriptive biases presented by various clinical trials are summarized in Table 4.

#### 4.3.1. Miscellaneous Effects in Healthy Subjects

De Liz et al. [159] performed a randomized crossover study to investigate the effects of açaí and juçara (*Euterpe edulis* Mart.) juices on biochemical and antioxidant parameters in humans. The participants were indicated to drink 200 mL/day of açaí juice for four weeks. The authors observed posttreatment improvement in HDL levels and antioxidant enzyme activities (SOD, CAT, and GPx) in their test subjects. However, there was a noteworthy limitation of this trial. Although this report indicated that the patients were asked about adverse events throughout the experimental process, no information was provided with regards to the results for this question.

Another trial showed that the consumption of 200 g of açaí pulp/day over a four-week timeframe afforded no modifications in total cholesterol, LDL and HDL, triglycerides, or apolipoprotein B (ApoB) in healthy female patients, besides the demonstration of significant antioxidant actions. Nonetheless, increased ApoA-I was observed, suggesting an improvement in the metabolism of this lipoprotein. The favorable actions on plasma HDL metabolism and antioxidant defenses indicate a potential use of açaí as an antilipidemic agent [178].

Additionally, Alqurashi et al. [179] investigated the effects of açaí intake on acute modifications in vascular function, along with a few other risk markers in healthy individuals. The authors observed improvement in vascular function. One limitation of this trial was the small number of patients. Furthermore, the authors did not include information about the gender of the participants.

Carvalho-Peixoto et al. [180] considered the effects of an açaí functional beverage on muscular and oxidative stress cardiorespiratory responses, biomarkers, perceived exertion, and time-to-exhaustion in healthy subjects during maximal treadmill running. Metabolic stress responses induced by exercise were found to be improved by açaí. In spite of this, this study was not double-blinded and used a small patient sample.

Gale et al. [181] performed a clinical trial to examine the effects of açaí on the hemodynamic and electrocardiographic parameters of healthy subjects. While a single-dose of a 500 mg gel capsule of açaí greatly reduced standing systolic blood pressure, it produced no other significant hemodynamic or electrocardiographic effects in volunteers in good health. However, this study did not include its randomization strategies or sample size estimation within its text, which prove to be limitations of the report.

#### 4.3.2. Auditory Disorder

A study performed by Oppitz et al. [31] revealed the beneficial impact of açaí on tinnitus. Specifically, açaí extract attenuates the discomfort associated with symptoms of tinnitus in human subjects. However, the number of patients considered in the study was small and some of the data analyses lacked complete clarity. Moreover, the authors of the paper failed to report any losses of follow-ups after the three months of treatment. While the authors described a relationship between açaí and levels of oxidative stress, discomfort, and anxiety caused by tinnitus, more data is needed.

#### 4.3.3. Effects on Bodyweight, Dyslipidemia and Metabolic Syndrome

Aranha et al. [142] conducted a randomized, double-blind, placebo-controlled clinical trial to evaluate the effects of a hypoenergetic diet and açaí consumption (200 g) in overweight patients with dyslipidemia and observed a reduction of oxidative stress and proinflammatory biomarkers in test subjects. After sixty days, the participants also presented significantly higher levels of vitamin A, although there was a decrease in vitamin E. Furthermore, IL-6 levels were noted to be significantly lower in the group exposed to açaí supplement, which, according to the report, was most likely due to the effect of anthocyanins. The authors described that the adverse effects to açaí, such as nausea, dyspepsia, constipation, diarrhea, or allergic reactions, were monitored during the trial. Nevertheless, the authors did not provide record of this information.

In addition, Kim et al. [182] evaluated the anti-inflammatory effect of açaí supplementation in patients diagnosed with MetS through a twelve-week randomized, double-blind, placebo-controlled clinical trial. However, the daily intake of 325 mL of an açaí-beverage (with 1139 mg/L of gallic acid) did not modify biomarkers for lipid and glucose metabolism in the study participants. Even so, açaí pulp juice significantly decreased IFN-γ levels in patients receiving the dietary açaí supplement. One limitation of this trial was the small number of included subjects and the lack of investigation of adverse outcomes among patients.

In overweight subjects, Udani et al. [183] revealed that the consistent intake of 100 g açaí pulp, twice a day for one month, reduced fasting glucose and insulin levels. Compared to baseline, the consumption of açaí improved the postprandial increase in glycemia following a standardized meal. These results also indicated that the consumption of 100 g of açaí pulp by overweight subjects engenders significant reductions in both total cholesterol and LDL levels.

#### 4.3.4. Effect on Prostate Cancer

Further, Kesller et al. [184] investigated the effects of a mixture of tea and açaí juice in patients with prostate cancer and observed a possible stabilization of prostate-specific antigen (PSA). Because the preparation of the dietary supplement used in this study involved many different components, a limitation of this study is the lack of ability to pinpoint the exact causative factor for the observed results.

## 5. Toxicity and Safety Studies

On the whole, current scientific literature is deficient in information regarding the toxicity of açaí. Marques et al. [18] published the first cytotoxic, genotoxic, and antigenotoxic assessment of açaí fruit oil in human cell cultures. These authors showed that the acute treatments of EOO (2.5, 10, 100, 500, and 1000 µg/mL) demonstrated neither cytotoxic effects nor DNA damage in HepG2 and human lymphocytes. Moreover, samples from mammalian leukocytes did not suffer any genotoxic effects following the administration of 1% of açaí oil at doses of 30, 100, and 300 mg/kg for 14 days.

Additionally, Ribeiro et al. [185] tested acute and subacute doses of açaí pulp in mice at 3.33, 10.0, and 16.67 g/kg and demonstrated that no genotoxic effects were induced by the açaí administration. To further illustrate this point, another study suggested the absence of toxic effects caused by açaí at acute doses up to 2000 mg/kg in animals (such as mice), which could be equated to the human consumption of 140 g of açaí at one time [10].

However, Marques et al. [186] used rat models to demonstrate that the oral administration of açaí oil at doses of 30, 100, and 300 mg/kg over a time period of 14 days resulted in altered thyroid cell follicular morphology and reduced size of follicular cells due to hypertrophy and unorganized growth. Interestingly, these doses of açaí oil also caused hepatocyte vacuolization, as well as a shift from eosinophilic to basophilic characteristics in the cells.

Moreover, Caiado et al. [187] explored the toxic effects of açaí fruit-based dye in the retina of rabbits. The results of this study revealed no potential toxicity at 10% and 25% concentrations of açaí dye. However, at a concentration of 35%, the açaí fruit-based dye induced ganglion cell edema 24 h after administration, as well as nerve bundle damage, multilamellar bodies, and vacuolization, in the retinal inner nuclear layers. While it can be seen that açaí is ordinarily safe, more data are needed in order to come to conclusively determine the toxicity of açaí.

## 6. Economic Importance

Açaí has enormous economic potential in many fields of industry. In the food industry, açaí may be used in the production many goods, ranging from probiotic beverages to desserts to ice cream. Due to its potent effects in preventing morphological changes in principally *Lactobacillus rhamnosus*, the fruit can be easily used as a suitable matrix for products that use this microorganism as a probiotic agent [188,189,190]. Additionally, açaí is an excellent alternative source of natural pigment for yogurts [191]. Of note, isotonic beverages were also developed based on the biological properties of açaí. Data have indicated a wider range of health benefits and antioxidant capacity in food formulations incorporating açaí than those of traditional commercialized isotonic drinks [192].

Moreover, in the cosmetic industry, açaí has been used to create multicomplex emulsions that enhance the photoprotective effects of sunscreens formulas. Due to its antioxidant capacity, açaí also has potential as an ingredient in various other skincare products, such as skin lotions [144,193,194]. Açaí has also been utilized as a stimulatory ingredient to enhance skin microcirculation in rat models [195]. Recently, Xiao et al. [196] developed and evaluated a new thermoreversible gel formulated with açaí extract. This study validated the implication of açaí extract in the treatment keratoconus through the use of a rabbit model, in which satisfactory therapeutic results were demonstrated.

In the biotechnological industry, açaí-derived ultrafine fibers have been used to compose innovative pH sensors for the visual monitoring of food quality [197]. Additionally, because açaí-derived biomass has been found to be capable of absorption of Cd (II), Pb (II), and Cr (III), it has been put to use in Brazil for the remediation of water pollution [198]. The residual biomass of açaí has also been processed and assessed for potential in energy production due to its physicochemical profile. Although açaí-derived energy is not highly generated yet, it is a prospective green alternative to the traditional globalized energy sources, such as the petroleum and the mineral coal [11]. All in all, it can be seen that there are multitudinous industrial purposes for açaí.

## 7. Conclusions, Limitations, and Future Perspectives

Our results show that açaí has medicinal properties and the economic potential for widespread use throughout the food and cosmetic industry. The fruit presents a rich phytochemical profile composed of phenolic compounds, quinones, terpenes, and norisoprenoids, all of which are related to its health-promoting and disease-preventing potential. In vitro and in vitro studies demonstrated that açaí possesses antioxidant and anti-inflammatory effects; exerts cardioprotective, gastroprotective, hepatoprotective, neuroprotective, and renoprotective activities; improves hyperinsulinemia and dyslipidemia; and shows antineoplastic actions. Additionally, açaí exerts antimicrobial and antiparasitic effects. Clinical trials have demonstrated that açaí protects against prostate cancer, MetS risk factors, and auditory dysfunctions. Moreover, its derivatives, such as berry extracts, whole fruit extracts, seed extracts, and phytochemically enriched extracts, have no hepatotoxicity, cardiotoxicity, or nephrotoxicity, strengthening its safety and health potential.

However, our work had some limitations. Firstly, numerous included studies did not specifically define the composition of the utilized açaí. Secondly, a limited number of clinical studies have been conducted on this fruit, especially concerning direct human consumption. In addition, the studies included in this paper are heterogeneous due to the variety of forms of açaí administration used (e.g., capsules, smoothie, juice, extract, mixture with other components) and in different doses. In short, the accumulated scientific evidence on the properties of açaí, in the absence of undesirable effects, awards this plant a promising future in health promotion and disease prevention. Moreover, there is a vast economic potential of açaí in the pharmaceutical, food, and cosmetic industries.

## Figures and Tables

**Table 1:** In vitro biological and pharmacological activities of various extracts and pure compounds of açaí tree.

Properties	Plant Part Used or Compounds	Models	Type of Extract	Concentrations	Observations	Reference
Antioxidant and anti-inflammatory	Açaí oil	Carrageenan-induced edematic mice paws, carrageenan-induced mice air pouches	EOO, EOO-βCD, and EOO-HPβCD	0.25, 0.5, 1.0, and 1.5 mg/mL	EOO-HPβCD achieved antioxidant activity 47% greater than that of the pure EOO	[47]
	Açaí seed extract	LPS-stimulated RAW 264.7 macrophages	Catechin-rich ethyl acetate açaí seed extract	125, 250, and 500 µg/mL	EO-ACET did not exert cytotoxic effects; the RAW macrophages showed lower levels of nitrite, IL-1β, IL-6, and IL-12	[48]
	Açaí extract made from skin and pulp fractions	RAW 264.7 macrophages treated with pro-inflammatory doses of OLZ	Hydroalcoholic	0.01, 0.05, 0.1, 1.0, 5.0, and 10 µg/mL	Açaí extract at 5 µg/mL showed reduction of NO, IL-1β, IL-6, TNF-α, and IFN-γ	[49]
	Açaí seed extract	RAW 264.7 macrophages	Açaí seed extract rich in flavan-3-ols	10, 30, 100, and 300 μg/mL	Açaí-treated macrophages presented lower NF-κB activation, TNF-α production, and oxidative stress	[50]
	Açaí berry freeze-dried extract	HepG2 cells	Hexane fraction, dried chloroform, dried butanol, and aqueous extracts rich in pheophorbides	50 μg/mL, 200 μg/mL, and 8.2 and 16.9 μM for pheophorbide A methyl ester and pheophorbide A, respectively	The methyl and ethyl esters of the common pheophorbide A parent demonstrated ARE-activation at 8.2 μM and 16.9 μM for pheophorbide A methyl ester and pheophorbide A, respectively	[51]
	Açaí seed extracts rich in phenolic bioactive compounds, especially (−)-epicatechin (497 mg/100 g), and (+)-catechin (403 mg/100 g)	HUVEC cells stressed by H_2_O_2_	Lyophilized	0.1–100 mg/mL for oxidative stress assays and 10 mg/mL for endothelial cell migration assays	Açaí prevented H_2_O_2_-cytotoxicity, oxidative stress, and migratory function loss, and stimulated the upregulation of Nrf2 antioxidant pathways via ERK	[52]
	Açaí berry extract	A liposome-rich environment with induced oxidation	Aqueous	50 mL	Açaí treatment protected the structures of a lipid-rich environment of liposomes from oxidative damage	[53]
	Açaí seed extract rich in a B-type (epi) catechin tetramer and procyanidin trimers	Human breast adenocarcinoma MCF-7 cells, non-small NCI-H460 cells lung cancer, cervical HeLa carcinoma cells, HepG2 cells, and non-tumor freshly porcine harvested cells	Aqueous	8 mg/mL for cytotoxicity screening and 1 mg/mL for antioxidant activity evaluation	Açaí aqueous seed extract had potent antioxidant capacity and exerted cytotoxic actions against HeLa cells	[54]
	Açaí fruit extract composed of 31.0 ± 2.4 mg/100 g of total anthocyanins	HUVEC cells and an *E. coli* bacteria strain	Aqueous	2.5 mg/mL for HUVEC cells and 100 mg/mL for *E. coli* bacteria	Açaí treatment blocked bacterial growth significantly; ROS production was limited and conferred protection against oxidative damage	[55]
	Freeze-dried açaí pulp powder rich in five different flavonoids: (2S,3S) dihyrokaempferol 3-*O* b-D-glucoside, (2R,3R) dihydrokaempferol 3 *O*-b-D-glucoside, isovitexin, velutin, and 5,40-dihydroxy-7,30,50 -trimethoxyflavone	RAW-blue cells induced by LPS	Flavonoid extracts/isolates	Velutin: 0.625, 1.25, 2.5, 5 μM; luteolin: 2.5, 5, 10, and 20 μM	Velutin exerted significant anti-inflammatory activities in SEAP assays; 5,40-dihydroxy-7,30,50 trimethoxyflavone demonstrated more potent antioxidant capacity compared to its isomer	[56]
	Antioxidant-rich fruit and berry juice blend of açaí as the predominant ingredient and other fruits and berries (white and purple grape, Nashi pear, acerola, aronia, cranberry, passionfruit, apricot, prune, kiwifruit, blueberry, wolfberry, pomegranate, lychee, camu camu, pear, banana), and bilberry with anthocyanins, predominantly cyanidin 3-rutoside, cyanidin 3-diglycoside, and cyanidin 3-glucoside	PMN cells, polymorphonuclear cells, and erythrocytes	MonaVie Active juice blend	Approximately 7.2 g of dissolved material	The blend protected erythrocytes from oxidative damage, prevented ROS production by polymorphonuclear cells, and reduced leukocyte migration through inhibition of FMLP	[57]
	Açaí berry pulp bioactive compounds	Human MCF-7 breast cancer cells stressed by H_2_O_2_	All bioactive compounds were extracted and isolated for the research procedures and analyzed by the results	Different concentrations of the isolated bioactive compounds	Açaí berry pulp bioactive compounds demonstrated high values in the OH radical scavenging assays	[58]
	Açaí fruit pulp and skin powder with 13.9 mg GAE/g of total polyphenolics	Human PMN cells	Acetone, water, and acetic acid extracts	5, 12.5, 25, 50, 125, 250, 500, and 1000 μg/mL	Açaí promoted high antioxidant capacity against the peroxyl radical and mild activity against peroxynitrite and hydroxyl radicals; inhibited COX-1 and COX-2	[59]
Antimicrobial	Açaí seed extracts rich in A- and B-type procyanidins	Human THP1 monocyte cells, monkey LLC-MK2 kidney epithelial cells, and HepG2 cells; *Staphylococcus aureus*, *Enterococcus faecalis*, *Escherichia coli*, *Salmonella typhimurium*, *Pseudomonas aeruginosa*, and *Candida albicans*	Hydroalcoholic and aqueous extracts	Mammalian THP1, LLC-MK2, and HepG2 cells were treated at 15.6–1000 μg/mL; microbial cells were treated at 2 mg/mL	Açaí extract exerted antimicrobial effects against Gram-positive bacteria and *Candida albicans* strains and was not cytotoxic to THP1 and LLC-MK2 mammalian cells; açaí also protected macrophages from ROS	[60]
	Dried açaí pulp powder extract	Erythrocytes from O+ individuals infected by chloroquine-sensitive and multidrug-resistant strains of *P. falciparum* and RAW 264.7 cells	Polyphenol-rich extracts: (1) rich in phenolic compounds, (2) rich in non-anthocyanin phenolics, and (3) rich in anthocyanins	Doses at concentrations ranging from 1.0 to 20.0 mg/L GAE	The açaí fraction rich in non-anthocyanin phenolics inhibited the growth of the parasites, and none of the fractions exerted cytotoxic effects in the cells	[61]
	Açaí pulp extract	HepG2 cells, planktonic cells, *Staphylococcus aureus*, and other Gram-positive bacteria	Methanolic extract of açaí pulp	The HepG2 cells were treated with 20 µL of the extract at 500-7.81 µg/mL; microbes were treated with açaí extract at concentrations ranging from 1 to 7.8 µg/mL	Açaí extract decreased the proliferation of cancerous HepG2 cells and inhibited biofilm production by planktonic cells and *Staphylococcus aureus* strains	[62]
	Açaí pulp, seed, and leaf extracts	*Clostridium perfringens*, *Staphylococcus aureus*, *Escherichia coli*, and *Pseudomonas aeruginosa*	Hydroalcoholic	10 μL of açaí extracts at concentrations ranging from 10 to 2.560 μg/mL	Açaí seed and pulp extracts showed significant inhibition against the proliferation of all investigated microorganisms	[63]
Neuroprotective	Açaí fresh fruits extract	Microglia EOC 13.31 cells line	Hydroalcoholic	The cells were treated with final concentrations ranging 0.001–1000 μg/mL	Açaí exposure reverted LPS-induced inflammation and ROS production and reduced cell proliferation induced by the LPS stress; reduced NLRP3, caspase-1, and IL-1β expression levels	[64]
	Açaí juice rich in orientin, homoorientin, taxifolin deoxyhexose, cyanidin 3-glucoside, and cyanidin 3-rutinoside	Neurons and astrocytes	Clarified	0–25% EO in Hank’s buffer at a final volume of 250 μL	Low concentrations of clarified açaí juice improved GABAergic neurotransmission by modulating GABA uptake	[65]
	Açaí fruit extract rich in anthocyanins (cyanidin 3-glucoside and cyanidin 3-rutinoside) and carotenoids (lutein, zeaxanthin, a-carotene, and b-carotene	Human neuroblastoma SH-SY5Y cells line	Hydroethanolic	0.5, 5.0, and 50 μg/mL	Açaí extract protected cells from 13% to 62% of the SY5Y cells from H_2_O_2_-related oxidative damage	[66]
	Freeze-dried açaí extracts rich in gallic acid, catechin, chlorogenic acid, caffeic acid, p-coumaric acid, epicatechin, orientin, vitexin, cyanidin-3-*O*-glucoside, luteolin, apigenin, and chrysin	Neuronal-like cells (SH-SY5Y) with mitochondrial complex I deficiency	Hydroalcoholic	The cells were treated with final concentrations ranging 0.001–1000 g/mL	Açaí significantly potentialized the expression of NDUFS7 and NDUFS8, augmenting the protein amount and enzyme activity of mitochondrial complex I and diminishing ROS production and lipid peroxidation	[67]
	Açaí berry extract	Immortalized DI TNC1 rat astrocytes stimulated by an Nrf2-ARE or an LPS-insulated NF-κB response element	Not reported	The cells were treated with final concentrations ranging from 6.25-50 μg/mL	Açaí inhibited the LPS-induced NF-κB reporter activity, as well as enhanced the antioxidant Nrf2/ARE response alone and also the Nrf2/ARE in the presence of the LPS-related stress	[68]
	Polyphenol-rich pulp extracts of açaí rich in cyanidin 3-*O*-glucoside, cyanidin 3-rutinoside, and delphinidin 3-glucoside	Sprague–Dawley rat embryonic hippocampal neuronal E18 cells and HT22 hippocampal cells	Aqueous	The cells were treated with final concentrations ranging 1–5 µg/mL	The treatment significantly caused a rapid recovery of the depolarized dopamine-(DA-)-induced Ca^2+^ influx neurons; there was attenuation in the inhibitor-induced autophagy dysfunction in the neurons	[69]
	Açaí fresh extract	Rat PC12 pheochromocytoma cells	Aqueous	The cells were treated with final concentrations ranging 0.5–50 µg/mL	The use of açaí was effective in preventing β-amyloid deposition in neuronal-like cells and further aggregation	[70]
	Pasteurized, freeze-dried açaí pulp extract rich in anthocyanins and other phenolic compounds. The study evaluated different fractions, such as ETOH, MEOH, ETAC, and ACE	Murine BV-2 microglial cells stressed by LPS treatment	Not reported	The cells were treated with final concentrations ranging 50 μg to 10 mg/mL	The treatment decreased nitrite production and iNOS expression by the ferulic acid content among the fractions. The MEOH, ETOH, and ACE fractions primarily exerted anti-inflammatory effects by downregulating COX-2, p38-MAPK, TNF-α, and NF-κB expressions	[71]
	Açaí fruit extract	Dissected cerebral cortex, cerebellum, and hippocampus of pretreated with H_2_O_2_ rats	Aqueous	The cells were treated with açaí pulp at a final concentration of 40% wt/vol	A negative correlation was observed between the açaí polyphenol content and the lipid and protein oxidative-related damage in the brain tissues	[72]
Antiadipogenic	Açaí seed extract rich in catechin and polymeric proanthocyanidins	3T3-L1 adipocytes	Not reported	0, 10, 25, 50, and 100 μg/mL	The extract inhibited adipogenesis by decreasing adipocyte differentiation through the decreasing expression of many adipogenic proteins and transcription factors of PPARɣ, SREBP-1, and FAS. Additionally, the extract suppressed lipid accumulation	[73]
	Frozen, concentrated, açaí juice rich in anthocyanins (cyanidin 3-glucoside and cyanidin-3-rutinoside) and flavonoids C glycosides (orientin, homoorientin, isovitexin, taxifolin deoxyhexose, and flavan-3-ol monomers)	3T3-L1 adipocytes	Not reported	The cells were differentiated with and without açaí polyphenols at concentrations of 2.5, 5, and 10 μg GAE/mL	The polyphenolic compounds reduced the intracellular lipid accumulation of adipocytes; downregulated PPARγ2 expression; and decreased the expression of adipogenic transcription factors, such as C/EPBα, C/EPBβ, Klf5, and SREBP-1c, and adipogenic genes, such as aP2, LPL, FATP1, and FAS	[74]
**Cardiovascular protective**	Açaí dietary powder supplement extract rich in anthocyanins (cyanidin-3-*O*-rutinoside) and flavonoids	HMEC-1 cells	Hydroethanolic	The cells were treated with final concentrations of 1–75 mg/L	Açaí powder exerted antiangiogenic effects without being cytotoxic and decreased the migration and invasion potentials of HMEC-1 cells, as well as the formation of capillary-like structures	[75]
**Anticancer**	Açaí pulp rich in anthocyanin cyanidin 3-rutinoside, (C3R, 214.09 ± 17.32 mg/100 g)	RKO human colon adenocarcinoma cells	Lyophilized	C3R at concentrations of 25, 50, and 100 μM.	C3R at concentrations of about 25 μM inhibited RKO cell motility, possibly exerting an anticancer potential	[76]
	Gold nanoparticles of açaí berries	Pancreatic (Panc-1) and prostate (PC-3) cancer cell lines	Aqueous	50-200 mg/mL of açaí berries extract and 0.0–0.4 mg/mL of açaí gold nanoparticles	The açaí gold nanoparticles showed potent anticancer activity against pancreatic and prostate cancer cell lines	[77]
	Kinetically stable açaí oil at a concentration of 50 mg oil/mL	Murine fibroblast NIH/3T3 normal cells and murine B16F10 melanoma cell lines	Nanoemulsion	PDT with the açaí oil nanoemulsion at 50 mg oil/mL concentration	Treated cells presented 85% of B16F10 melanoma cell lines death by apoptosis while preserving NIH/3T3 normal cell viability	[78]
	Açaí seed hexane, chloroform, and ethyl acetate extract fractions	Human MCF-7 breast adenocarcinoma-derived cells	Hydroalcoholic	The cells were treated with final concentrations ranging from 10, 20, 40, and 60 μg/mL	The results showed that the ethyl acetate fraction most effectively reduced MCF-7 cell viability by causing necroptosis	[79]
	Bark, seed, and total açaí fruit extracts	Human Caco-2 and HT-29 colon adenocarcinoma cells and human MDA-MB-468 and MCF-7 mammary adenocarcinoma cells	Hydroalcoholic	10, 20, and 40 μg/mL	Only MCF-7 cells responded to the açaí treatment; the extracts reduced cell viability and altered cell morphology	[80]
	Frozen, concentrated, clarified açaí juice	Nonmalignant CCD-18 colon fibroblast cells and malignant colon cancer HT-29 and SW-480 cells	Polyphenolic extract	Doses ranging from 5–20 mg/L	Açaí inhibited the growth of SW-480 cells with no cytotoxic effects against CCD-18 cells. Prooncogenic proteins were downregulated, as well as Sp-targets Bcl-2, the vascular endothelial growth factor, and the factor survivin	[81]
	Monomeric (cyanidin-3-rutinoside and cyanidin-3-glucoside) and polymeric (mixture of anthocyanin adducts) anthocyanin fractions from açaí fruit	Human HT-29 colon adenocarcinoma cells and colon Caco-2 carcinoma cells	Anthocyanin extracts	Doses ranging from 0.5 to 100 μg cyanidin-3 glucoside equivalents/ml	Açaí anthocyanins inhibited colon HT-29 cancer cell proliferation (95.2%)	[82]
	Anthocyanin-rich extract from açaí (312 mg of GAE/g, 124 mg RE (flavonoid content), and 100 mg CGE (anthocyanin content))	Rat C-6 brain glioma cells and human MDA-468 breast cancer cells	Lyophilized	50, 100, and 200 μg/mL	Açaí suppressed the proliferation of rat C-6 brain glioma cells but did not affect human MDA-468 breast cancer cells	[83]
	Açaí juice	XV 185-14c strain of *Saccharomyces cerevisiae*	Not reported	5%, 10%, and 15% wt/vol	The use of the açaí in higher concentrations demonstrated mutagenic effects	[84]
	Açaí pulp extract divided into whole pulp fraction, lipophilic fraction, C18 bound phenolics and anthocyanins fraction, ethyl acetate soluble polyphenolics, isolated anthocyanins fraction, C18 non-retained fraction, C18 bound phenolics and anthocyanins fraction, hydrolyzed anthocyanins fraction, and hydrolyzed ethyl acetate soluble polyphenolics fraction	Human HL-60 leukemia cells	Not reported	Cells were treated with all açaí fractions at concentrations ranging from 0.0–10.7 µM	The polyphenolic fraction decreased cell proliferation from 56 to 86% and increased cell apoptosis due to the caspase-3 activation pathway	[85]
	Freeze-dried açaí pulp	Not reported	In vitro digested freeze-dried açaí pulp	1 g of the digested açaí pulp	In the feces examination, the pulp decreased the number of the *Bacteroides–Prevotella* spp. and *Clostridium histolyticum* colonies	[86]
	Açaí berry pulp and oil extract rich in phenolic acids (protocatechuic acid, *p*-hydroxybenzoic acid, vanillic acid, syringic acid, ferulic acid, catechin, and epicatechin), flavonoids, and procyanidins	Human HT-29 colon adenocarcinoma cells	Polyphenolic extracts	The cells were treated with polyphenolics ranging the final concentrations of 0.04–12 µg of GAE/mL	The treatment could effectively inhibit cellular proliferation by up to 90.7%	[87]
Musculoskeletal health	Velutin, a bioactive compound of açaí	RAW 264.7 osteoclast precursor cell line stimulated with RANKL	Not reported	The cells were treated with final concentrations ranging 0.5–2.0 μM	Velutin was not cytotoxic to RAW 264.7 osteoclast or undifferentiated cells, reduced osteoclast differentiation, and exerted potential anti-inflammatory effects downregulating the HIF-1α production	[88]
	Dried açaí berry powder extract	RAW 264.7 cells stimulated with RANKL	Not reported	The cells were treated with final concentrations ranging the doses 25–100 µg/mL	Açaí decreased IL-6 and TFN-α and showed inhibitory actions of osteoclastogenesis and osteoclastic activity. There was an increase in IL-3, IL-4, and IL-13	[89]

Abbreviations: ACE: acetone; aP2: adipocyte fatty acid-binding protein 2; ARE: antioxidant response element; ATP: adenosine triphosphate; Bcl-2: B-cell lymphoma 2; beta-amyloid: (Aβ); BrdU: 50-bromodeoxyuridine; CAP-e: cell-based antioxidant protection of erythrocytes; CAT: catalase; C/EBPα: CCAAT/enhancer binding protein alfa; C/EBPβ: CCAAT/enhancer binding protein beta; CGE: cyanidin 3-glycoside equivalents; COX-1: cyclooxigensase-1; COX-2: cyclooxygenase-2; C3R: anthocyanin cyanidin 3-rutinoside; DCFH-DA: fluorescent dichlorofluorescein diacetate; EO-ACET: *E. oleracea* ethyl acetate extract; EOO: *E. oleracea* oil; EOO-HPβCD: *E. oleracea* oil with hydroxypropyl-β-cyclodextrin complex; EOO-βCD: *E. oleracea* oil with hydroxypropyl-β-cyclodextrin; ETAC: ethyl acetate; ERK: extracellular signal regulated kinases; ETOH: ethanol; FAS: fatty acid synthase; FAS: fatty acid synthase; FATP1: fatty acid transport proteins; FMLP: bacterial peptide f-Met-Leu-Phe; GABA: γ-aminobutyric acid; GAE: gallic acid equivalents; HIF-1α: hypoxia-inducible factor-1α; HMEC-1: human microvascular endothelial cells; HPβCD: hydroxypropyl-β-cyclodextrin complex obtained by kneading; HUVEC: immortalized human umbilical vein endothelial cells; H_2_O_2_: hydrogen peroxide; ICAM-1: intracellular adhesion molecule-1; IFN-γ: interferon-γ; IL-3: interleukin-3; IL-4: interleukin-4; IL-6: interleukin-6; IL-8: interleukin-8; IL-12: interleukin-12; IL-13: interleukin-13; IL-1β: interleukin-1β; iNOS: nitric oxide synthase; Klf5: transcription of Kruppel-like factor; LPL: lipoprotein lipase; MTT: thiazolyl blue tetrazolium bromide; LLC-MK2: monkey kidney epithelial cells; LPS: lipopolysaccharide; MDC: labeling of autophagic vacuoles with monodansylcadaverine; MEOH: methanol; mTOR: mammalian target of rapamycin; NF-κB: nuclear factor κB; NLRP3: NOD-like receptor pyrin-domain containing 3; NO: nitric oxide; NOD: nucleotide oligomerization domain; Nrf2: nuclear factor-erythroid factor 2-related factor 2; Nrf2-ARE: nuclear factor-erythroid factor 2-related factor 2/antioxidant response element; OH: hydroxyl; OLZ: olanzapine; ORAC: oxygen radial absorbance capacity; OXPHOS: analysis of human mitochondrial oxidative phosphorylation; PDT: photodynamic therapy; PPARɣ: peroxisome proliferator-activated receptor ɣ; p38-MAPK: p38 mitogen-activated protein kinase; RANKL: receptor activator of nuclear factor-κB ligand; RE: rutin equivalents; RP: reducing power; SEAP: secreted embryonic alkaline phosphatase; SOD: superoxide dismutase; SREBP-1: sterol regulatory element binding protein-1; SREBP-1c: sterol regulatory element binding protein-1c; TAC: total antioxidant capacity; TAO: total antioxidants; THP1: human monocyte cells; ThT: thioflavin t; TNF-α: tumor necrosis factor-α; VCAM-1: vascular cell adhesion molecule-1; βCD: β-cyclodextrin; [3H]TBOB: [3H]-t-butylbicycloorthobenzoate.

**Table 2:** In vivo biological and pharmacological activities of various extracts and pure compounds of açaí tree.

Properties	Plant Part or Active Compounds	Type of Extraction	Experimental Model	Dose	Route of Administration	Observations	Reference
Antioxidant	Açaí pulp with 549.5 mg/100 g of gallic acid equivalent	The açaí pulp was purchased commercially and stored	Female Fischer rats	Diet supplemented with 2% of açaí pulp	Oral by feeding	The açaí pulp supplemented diet augmented antioxidant GPx-1, GPx-4, and SOD1 mRNA genetic expression in the liver	[90]
	Açaí pulp	The açaí pulp was purchased commercially and stored	Male Wistar rats	Diet supplemented with 5% of açaí pulp	Oral by feeding	Açaí supplementation reduced oxidative stress and improved energetic metabolism	[91]
	Açaí seed extract	The açaí was acquired and the extract was made in a laboratory	Male Wistar rats	Doses at concentrations of 100 mg/mL 200 mg/mL	Intragastric gavage	The açaí seed extract did not diminish the cachectic syndrome in a rat model of tumorigenesis	[92]
	Pasteurized açaí pulp with a high capacity for neutralizing free radicals	The açaí pulp was purchased commercially and stored	Female Fisher rats	Diet supplemented with 2% of açaí pulp	Oral by feeding	Açaí pulp could effectively control the oxidative species production by neutrophils and increased liver antioxidant defenses	[93]
Anti-inflammatory	Açaí oil	The açaí oil was purchased commercially and stored	Male Wistar albino rats and male Swiss albino rats	Doses of 500, 1000, and 1500 mg/kg	Orally	The anti-inflammatory effects were associated with prostaglandin synthesis inhibition	[19]
	Açaí stone extract	The açaí berries were obtained, and the stone extract was made and stored	Eight-week-old male mice	300 mg/kg/day	Intragastric gavage	The supplementation with açaí seed extract could significantly reduce inflammatory and oxidative responses	[94]
Analgesic	Açaí stones extract rich in proanthocyanidins	Hydroalcoholic extract	Male Swiss mice	The açaí stone extract was dissolved in distilled water at a concentration of 10 mg/mL	Intragastric gavage	The extract exerted antinociceptive effects	[95]
Antimicrobial	Açaí fractions	Rich polyphenol fractions of açaí	Murine models infected with P. chabaud	Doses of 10, 15, and 20 mg/kg/day of the açaí polyphenol-rich fractions	Intragastric gavage	The higher doses of açaí fractions reduced parasitemia and increased the survival rates of infected animals	[61]
Gastroprotective	Açaí seed extract with considerable amounts of proanthocyanidins and lesser amounts of catechin and epicatechin	Hydroalcoholic	Male Wistar rats	Doses of 10, 30, and 100 mg/kg	Orally	A higher dose significantly reduced inflammation, oxidative stress, and macroscopy and histological parameters of the colitis	[12]
	Açaí berries dried extract with high radical scavenger capacity	Dried extract	Female Wistar rats	Doses of 30 and 100 mg/kg (PO) and 3 mg/kg (IP)	Orally or intraperitoneally	The extract reduced inflammation and maintained oxidative balance in the gastric mucosa	[96]
Neuroprotective	Clarified açaí juice containing no lipids, proteins, or fibers	Microfiltrated and centrifugated açaí juice	Male Swiss mice	Doses of 10 µL/g	Intragastric gavage	The use of açaí clarified juice effectively protected the brain against oxidative stress in specific areas related to convulsive crises	[97]
	Açaí seeds extract 88% of proanthocyanidins	Aqueous extract	Male Wistar rats	200 mg/kg/day	Intragastric gavage	Açaí exerted anti-anxiety effects by reducing hypothalamus–pituitary–adrenal axis reactivity to stress and increasing the NO–BDNF–TRKB pathway	[98]
	Fresh açaí extract	Fresh herbal capsules	Male Wistar albino rats	Doses of 100 mg/kg/day or 300 mg/kg/day	Intragastric gavage	Açaí did not improve learning and memory abilities	[99]
	Açaí frozen pulp	The frozen açaí pulp was purchased commercially and stored	Male Wistar rats	7 μL/g/day	Intragastric gavage	The açaí frozen pulp exerted antioxidant effects on the brain of the rats	[100]
	Lyophilized açaí pulp	The lyophilized açaí pulp was purchased commercially and stored	Aged male Fischer 344 rats	A diet containing 2% of açaí pulp	Oral by feeding	The supplementation conserved the memory of rats due to the anti-inflammatory and antioxidant effects of açaí berry	[101]
	Açaí frozen pulp	The frozen açaí pulp was purchased commercially and stored	Male Wistar rats	Dose of 7 μL/g	Intragastric gavage	The use of açaí prevented an increase in IL-1β, IL-18, and TNF-α, while IL-6 and IL-10 levels remained unchanged	[102]
	Açaí frozen pulp with 1.19 ± 0.20 mg/100 g of catechin	The frozen açaí pulp was purchased commercially and stored	Wistar rats	Açaí pulp was diluted in distilled water at a concentration of 40% wt/vol	Orally	Açaí exerted antioxidant effects against neurodegenerative diseases in a rat model of hydrogen peroxide-induced nervous damage	[72]
	Freeze-dried açaí powder	The freeze-dried açaí powder was purchased commercially and stored	Male Fischer rats	2% of the freeze-dried açaí powder	Oral by feeding	The freeze-dried açaí powder modulated the Nrf2 pathway and protected neuronal cells against ubiquitin–proteasomal degradation	[103]
	Clarified açaí juice containing no lipids, proteins, or fibers, but with >1400 mg GAE/L	Microfiltrated and centrifugated açaí juice	Male Swiss mice	10 µL/g	Intragastric gavage	The treatment effectively abolished despair-like and anhedonia behaviors and protected the hippocampus, striatum, and prefrontal cortex from oxidative damage related to depression	[104]
Antilipidemic	Açaí oil	The açaí oil was purchased commercially and stored	Male Wistar rats	1226 mg/kg/day	Orally	The results suggested that the use of açaí oil was effective in reducing atherosclerosis in rats with dyslipidemia	[105]
	Açaí oil	The açaí oil was purchased commercially and stored	Male Wistar rats	1226 mg/kg/day	Intragastric gavage	The açaí oil was able to antagonize cholesterol and triglycerides increases among rats	[106]
	Pasteurized açaí pulp	The frozen açaí pulp was purchased commercially and stored	Female Fischer rats	Standard or a high-fat diet supplemented with 2% of açaí pulp	Orally by feeding	The supplementation promoted an anticholesterolemic effect by increasing the expression of subfamily G transporters, ATP-binding cassette, and LDL-R genes	[107]
	Pasteurized açaí pulp	The frozen açaí pulp was purchased commercially and stored	Female Fischer rats	Standard and a high-fat diet supplemented with 2% of açaí pulp	Oral by feeding	The açaí pulp supplementation improved antioxidant status and diminished cholesterol serum levels	[36]
	Açaí seed flour	The açaí flour was purchased commercially and stored	Male C57BL/6 mice	Diet supplemented with 15% or 30% of açaí flour	Oral by feeding	Açaí flour increased cholesterol excretion among mice fed a high-fat diet and prevented the development of obesity and NAFLD	[108]
	Fresh açaí berries extract	The açaí berries were obtained and stored for further aqueous extract production	Male New Zealand rabbits	80 mL of fresh açaí extract was dissolved in water	Oral by drinking water	Fresh açaí berries extract significantly improved the lipid profile and the atherosclerosis statuses in an atherosclerosis-induced rabbit model	[109]
Hepatoprotective	Açaí pulp with 549.5 mg GAE/100 g of polyphenols	The açaí pulp was purchased commercially and stored	Female Fisher rats	Standard and high-fat chow with 2% of açaí pulp	Oral by feeding	The supplementation had protective effects in dams against NAFLD and protected the offspring from the effects of a maternal high-fat diet with lipid excess	[110]
	Lyophilized açaí pulp	The açaí pulp was purchased commercially and stored	Male Fischer rats	Standard chow with 2% of the lyophilized açaí pulp	Oral by feeding	The lyophilized açaí pulp diminished inflammation and reduced liver steatosis	[111]
	Açaí pulp with 0.035 g/100 g of procyanidin	The açaí pulp was purchased commercially and stored	Wistar rats	1 mL/100 g	Intragastric gavage	The treatment reduced alcohol-induced liver injury in rats by diminishing inflammation and oxidative stress	[112]
	Açaí seed extract with 265 mg/g of polyphenols	Hydroalcoholic extract	Male Wistar rats	200 mg/kg/day	Intragastric gavage	The extract, in conjunction with exercise training, decreased glucose and lipid serum levels, serum hepatic enzymes, and liver triglycerides	[113]
	Filtered açaí pulp with 458.6 mg GAE/100 g of polyphenols and 13.59 mg/100 g of monomeric anthocyanins	The açaí oil was purchased commercially and stored	Female Fischer rats	2 g/day	Intragastric gavage	The açaí supplementation protected liver steatosis and injuries in a high-fat diet-rats	[114]
	Açaí water extract	The açaí pulp was obtained commercially and stored for future aqueous extract preparation	Male Swiss mice	3 g/kg/day	Intragastric gavage	The extract prevented liver damage, attenuated inflammation, and decreased oxidative stress	[115]
	Açaí water extract with 118.13 mg GAE/100 g of phenolic compounds and 9.23 mg/100 g of flavonoid compounds	The açaí pulp was obtained commercially and stored for future aqueous extract preparation	Male Swiss mice	3 g/kg/day	Intragastric gavage	The use of açaí increased the production and effectiveness of adiponectin, improving insulin sensitivity and increasing PPAR-α-mediated fatty acid oxidation	[116]
	Açaí seeds extract rich in catechin and epicatechin	Hydroalcoholic	Male C57BL/6 mice	300 mg/kg/day	Intragastric gavage	The use of the extract significantly reduced obesity and hepatic steatosis	[35]
Antidiabetic	Açaí seed extract with 265 mg/g of polyphenols	Hydroalcoholic extract	Male Wistar rats	200 mg/kg/day	Intragastric gavage	The extract exerted an antidiabetic effect in the diabetic-induced rats by potentializing the insulin-signaling pathway in skeletal muscles cells and adipose tissue, increasing GLP-1 levels	[117]
Antihypertensive	Açaí stones extract with 265 mg/g of polyphenols	Hydroalcoholic	Male Wistar rats	200 mg/kg/day	Orally	The supplementation with açaí protected against vascular changes and endothelial dysfunction due to antihypertensive and antioxidant effects	[118]
	Açaí seed extract with high amounts of proanthocyanidins	Hydroalcoholic	Female Wistar rats	200 mg/kg/day	Oral by drinking water	The açaí seed extract protected against cardiovascular changes and intrauterine growth restriction	[22]
	Açaí seed extract with 265 mg/g of phenolic compounds	Hydroalcoholic	Female Wistar rats	200 mg/kg	Intragastric gavage	Açaí promoted vasodilator and antioxidant effects	[119]
Cardioprotective	Açaí pulp with 170 mg/100 g of gallic acid and 15.6 mg/100 g of total anthocyanins	The açaí pulp was purchased commercially and stored at −80 °C for later use in standard chow	Male Wistar rats	Standard chow with 2% and 5% of açaí pulp	Orally by feeding	Supplementation with açaí pulp attenuated cardiac remodeling after myocardial infarction.	[6]
	Açaí seed extract	Aqueous	Male Wistar rats	Açaí seed extract in a dose of 200 mg/kg/day	Orally by drinking water	Reduced SBP, restored of endothelial and renal functions, decreased inflammation and oxidative stress, and attenuated of the endothelial dysfunction	[120]
	Lyophilized açaí pulp with 3300 mg/100 g of total polyphenols and 6.45 to 31.0 mg/100 g of anthocyanins	The açaí pulp was purchased commercially and stored	Male Fischer rats	High-fat diet supplemented with 1% of the lyophilized açaí pulp	Orally by feeding	Açaí supplementation may decrease cardiac remodeling and increase cardiac function	[121]
	Açaí pulp extract	Aqueous extract	Male Wistar rats	100 mg/kg and 300 mg/kg	Intravenous	There were elevations in acute blood flow induced by açaí extract	[122]
	Açaí pulp	The açaí pulp was purchased commercially and stored	Male Wistar rats	Standard chow with 5% of açaí pulp	Oral by feeding	The supplementation reduced left ventricular dysfunction, oxidative stress, changes in the myocardium metabolism, and MMP-2 activation	[123]
	Açaí seed extract	Hydroalcoholic	Male Wistar rats	100 mg/kg/day	Intragastric gavage	Açaí prevented the development of exercise intolerance, cardiac fibrosis, cardiac dysfunction, and cardiac hypertrophy	[124]
	Açaí seed extract	Hydroalcoholic	Young male Wistar rats and spontaneously hypertensive rats	200 mg/kg/day	Orally	Açaí seed extract prevented vascular remodeling and decreased the percentage of elastic fibers, media/lumen ratio, hypertension, and oxidative damage	[125]
Renoprotective	Açaí seed extract with 265 mg/g of polyphenols	Hydroalcoholic lyophilized extract	Male Wistar rats	200 mg/kg/day	Orally by drinking water	The extract significantly reduced renal injury and prevented renal dysfunction	[126]
	Açaí seed extract with 265 mg/g of polyphenols	Lyophilized açaí seed extract	Male Wistar rats	200 mg/kg/day	Orally	The açaí seed extract exerted renoprotective effects, diminished renal injury, and prevented renal dysfunction	[127]
	Açaí berry extract	Not reported	Male Wistar albino rats	Doses of 100 and 200 mg/kg/day	Orally	The extract was capable of attenuating renal damage	[128]
	Açaí fruit extract	Not reported	Male Wistar albino rats	Doses of 500 and 1000 mg/kg)	Intragastric gavage	The açaí fruit extract ameliorated the ischemia–reperfusion kidney-induced syndrome bilaterally in a dose-dependent manner	[129]
Anticancer	Lyophilized açaí pulp with 214.09 ± 17.32 mg/100 g of cyanidin 3-rutinoside and 1908.5 ± 24.4 mg/100 g of β-carotene	The lyophilized açaí pulp was purchased commercially and stored	Male Wistar rats	Standard chow with 5% or 7.5% of the lyophilized açaí pulp	Oral by feeding	The pulp exerted potential antitumor activity	[76]
	Açaí fruit extract	Hydroalcoholic extract	Female Wistar rats	200 mg/kg	Intragastric gavage	The extract promoted anti-inflammatory and antiangiogenic effects	[130]
	Spray-dried açaí powder	Açaí pulp was purchased commercially and dried to be sprayed	Male Wistar rats	A diet containing 5% of spray-dried açaí powder	Oral by feeding	The results showed that spray-dried açaí powder could effectively reduce the development of chemically-induced carcinogenesis	[131]
	Açaí pulp powder with 0.5% of polyphenolic content and freeze-dried açaí powder	The açaí pulp was purchased commercially and transformed into powder, then and the freeze-dried product was stored	Azoxymethane/dextran sulfate sodium-treated mice	0.5 g/5 mL of phosphate-buffered saline was administered as pellets containing 5% of açaí powder	Orally	The use of açaí protected the mice model of colon tumorigenesis against cancer development	[132]
	Spray-dried açaí fruit pulp containing high amounts of anthocyanins (cyanidin 3-glucoside and cyanidin 3-rutinoside) and carotenoids (lutein, α-carotene, β-carotene, and 9-cis β-carotene)	The açaí pulp was dried and stored	Male Swiss albino mice	A low-fat diet containing 2.5% or 5.0% of açaí fruit pulp powder	Oral by feeding	The use of açaí attenuated carcinogenesis principally by increasing antioxidant glutathione capacity and attenuating DNA damage	[133]
	Kinetically stable açaí oil nanoemulsion in a concentration of 50 mg oil/mL	Nanodroplets	C57BL/6 female mice	Rats were treated five times with nanodroplets containing the nanoemulsion with 50 mg of açaí oil/mL	Nanodroplets, orally	The açaí oil nanodroplets showed a significant reduction in the tumor volume	[78]
	Açaí flakes extract	Dehydration of açaí berries	Male F344 rats	Diet containing 5% berry flakes	Oral by feeding	The flakes exerted inhibitory effects on esophagus tumor progression	[134]
Wound-healing	Açaí berry extract	Aqueous extract	Sprague–Dawley rats	Treatments with 1%, 3%, or 5% of açaí berries aqueous extract were conducted	Application on lesions	The extract was not cytotoxic and significantly increased fibroblast migration and fibronectin expression	[135]
	Açaí berry extract	Aqueous extract	Sprague–Dawley rats	Treatments with 1%, 3%, or 5% of açaí berries aqueous extract were conducted	Application on oral lesions	The use of açaí extract significantly improved the healing progress in wounds of rats’ oral mucosa	[136]
Miscellaneous effects	Extract of açaí seeds with 25.12 mg/g of polyphenols, 9.048 mg/g of CAE, 0.258 mg/g of MRE, and 9.798 mg/g of CE	Ethanol extract	Male Wistar rats	Doses of 200 mg/kg, 300 mg/kg, and 400 mg	Intraperitoneal	The açaí extract demonstrated myorelaxant activities in the animals	[137]
	Açaí seeds extract with 265 mg/g of polyphenols	Hydroalcoholic	Male Wistar rats	200 mg/kg/day	Intragastric gavage	The extract improved the aerobic physical performance (↑ vascular function), reduced oxidative stress, and upregulated mitochondrial biogenesis key proteins	[138]
	Açaí fruit extract	Hydroalcoholic	Female Sprague–Dawley rats	200 mg/kg/day	Intragastric gavage	The extract significantly suppressed the establishment and growth of endometriosis	[139]
	Dried açaí	Açaí-enriched diet	Male Wistar rats	The dried açaí was mixed with the standard diet but was not calculated	Oral by feeding	The açaí-supplemented diet exerted eye protection and antioxidant effects	[140]
	Açaí extract	Not reported	C57BL/6NCrSlc mice	10 mL/kg/day	Intragastric gavage	The use of açaí can stimulate erythropoietin production by inducing a hypoxic renal condition	[141]

Abbreviations: ↑: increase; ATP: adenosine tri-phosphate; CAE: catechin equivalents; CAT: catalase; CE: cyanidin equivalents; CO: carbonyl protein; COX-2: cyclooxygenase-2; DMBA: 7,12-dimethylbenzanthracene; eNOS: endothelial nitric oxide synthase; GAE: gallic acid equivalent; GLP-1: glucagon-like peptide 1; GPx: glutathione peroxidase; HDL: high-density lipoprotein cholesterol; HOMA-IR: homeostatic model for insulin resistance assessment; IL-1β: interleukin-1β; IL-6: interleukin-6; IL-10: interleukin-10; IL-18: interleukin-18; LDL: low-density lipoprotein cholesterol; LDL-R: low-density lipoprotein receptor; MAB: mesenteric arterial bed; MDA: malonaldehyde; MMP-2: metalloproteinase-2; MPO: myeloperoxidase; MRE: myricetin-3-O-α-L-rhamnopyranoside equivalents; mRNA: RNA messenger; NAFLD: nonalcoholic fatty liver disease; NF-ĸB: nuclear factor-κB; NO-BDNF-TRKB: nitric oxide-brain-derived neurotrophic factor- tropomyosin receptor kinase B; NOS: nitric oxide synthase; Nrf2: nuclear factor erythroid 2-related factor 2; ROS: reactive oxygen species; RT–PCR: real-time quantitative reverse transcription–polymerase chain reaction; SBP: systolic blood pressure; SOD: superoxide dismutase; TC: total cholesterol; TG: triglycerides; TLR 4: toll-like receptor 4; TNF-α: tumor necrosis factor-α; VEGF: vascular endothelial growth factor; VLDL: very-low-density lipoprotein cholesterol.

**Table 3:** Clinical trials showing the effects of açaí on human health.

Type of the Study and Patients	Interventions	Outcomes	Adverse Effects	Reference
Healthy subjects				
Randomized crossover study with 38 healthy adults (22♀, 16♂; 19–48 y) in Brazil	Participants received 200 mL/day of açaí (*n* = 19) or juçara (*Euterpe edulis*) juice (*n* = 19) for 4 weeks with a 4-week wash-out period	No modifications in glycemia or lipid profile before the treatment period but improvement of TAC, OSI, CAT, and GPx levels	Not reported by the authors	[159]
40 healthy women (24 ± 3 y).	200 g of açai pulp/day for 4 weeks	No modifications in anthropometric parameters, arterial pressure, glucose, insulin, LDL, and HDL, triglycerides, and ApoB; increase of ApoA-I and TAC	Not reported by the authors	[178]
Randomized, double-blind, crossover-controlled trial with 23 healthy males (30–65 y) with a BMI 25–30 in the United Kingdom	Consumption of an açaí-based smoothie (694 mg total phenolics) or a macronutrient-matched control smoothie with a high-fat breakfast meal modification	Improvement of vascular function (increases in flow-mediated dilatation compared to control (*p* = 0.001). A significant reduction of iAUC for total peroxide oxidative status after açaí intake. No significant modifications for heart rate, blood pressure, or postprandial glycemia	Patients did not report AE	[179]
Simple-blinded randomized intervention trial with fourteen male athletes (mean age of 26 y) in Brazil	Performance of 3 tests: 45 a ramp-incremental maximal test and two maximal bouts in two conditions (açaí or control) at 90% VO^2^ max. After the first exercise bout, subjects drank 300 mL of freeze-dried and were instructed to intake the fruit 3 consecutive days, 1 h before the exercise bout to exhaustion	Increase in time to exhaustion during short-term high-intensity (*p* = 0.045), attenuating the metabolic stress induced by exercise	Not reported by the authors	[180]
Randomized, double-blind, placebo-controlled crossover study with 20 participants (13♀, 7♂; 22.4 ± 2.50 y) in the USA	Phase 1: subjects received two capsules (500 mg of açaí or placebo). After a 7-day wash-out, subjects returned for phase 2 and consumed the opposing treatment	After the first dose, no significant differences for ECG between groups, and no differences were seen for the primary or secondary hemodynamic endpoints (except for significant lower systolic blood pressure at 6 h with açaí)	Patients did not report AE	[181]
Auditory disorder				
Randomized, double-blind study with 30 patients (14♀, 16♂; mean age of 50.5 y); complaint of tinnitus, hearing thresholds; annoyance score of at least four in Brazil	Patients were divided into a placebo (starch capsules) and a treated group that received an extract of dry açaí (100 mg/capsule)	Reduction in the discomfort of tinnitus evaluated by THI (*p* = 0.006); significant improvement for anxiety disorders symptoms (*p* = 0.016). No significant differences for oxidative metabolism biomarkers, but a decrease in posttreatment values for all groups	Patients did not report AE	[31]
Overweight, dyslipidemia, and metabolic syndrome				
Randomized, double-blind, placebo-controlled clinical trial with 69 subjects (BMI > 25 kg/m^2^) (46♀, 23♂; 20–59 y) in Brazil	Participants with at least one lipid profile alteration that received 200 g of açaí or placebo, and a hypo-energetic diet (calculated individually)/60 days	Reduction of oxidative stress and improvement of inflammatory status (decrease of IL-6 and INF-γ)	Not reported by the authors	[142]
Randomized, double-blinded, and placebo-controlled trial with 37 subjects with MetS (BMI 33.5 ± 6.7 kg/m^2^; 26♀, 11♂; 18–65 y) in USA	Intake of 325 mL of açaí beverage twice/day or placebo/12 weeks	No modifications on lipid and glycemic profile; significant reduction of IFN-γ and urinary levels of 8-isoprostane, compared to the placebo group (*p* = 0.0141 and 0.0099, respectively)	Not reported by the authors	[182]
Open label pilot study with 10 adults, 18–65 y (BMI ≥ 25 kg/m^2^ and ≤30 kg/ m^2^) in USA	Intake of 100 g açai pulp twice daily for 1 month	Reductions in serum blood glucose and insulin levels (*p* < 0.02), CT (*p* = 0.03), LDL. No effects on blood pressure, CRP or NO metabolites	Patients did not report AE	[183]
Prostate cancer				
Phase II, Simon 2-stage clinical trial in subjects showing biochemically recurrent prostate cancer (54–80 y) USA	Subjects received açaí juice product (mix of fruit juices and tea extracts, with 80% of the juice produced with açaí berry), twice daily, for 36 weeks	PSA response >50% was observed in 1/21 subjects within 30 w of the treatment. The PSA doubling time was lengthened in most patients (71%)	Patients did not report AE	[184]

Abbreviations: AE: adverse effects; BMI: body mass index; CAT: catalase; CRP: C-reactive protein; ECG: electrocardiographic; GPx: glutathione peroxidase; iAUC: incremental area under the curve; HDL: high density lipoprotein; IFN-γ: interferon-γ; LDL: low density lipoprotein; MetS: metabolic syndrome; NO: nitric oxide; OSI: oxidative stress index; PAC: plasma antioxidant capacity; PSA; prostate specific antigen; ROS: reactive oxygen species; TAC: total antioxidant capacity; TC: total cholesterol; THI: Tinnitus Handicap Inventory.

**Table 4:** Descriptive biases presented by the included clinical trials performed with açaí.

Study	Question Focus	Appropriate Randomization	Allocation Blinding	Double-Blind	Losses (<20%)	Prognostic and Demographic Characteristics	Outcomes	Intention to Treat Analysis	Sample Calculation	Adequate Follow-Up
Oppitz et al. [31]	Yes	Yes	Yes	Yes	Yes	Yes	Yes	Yes	Yes	Yes
de Liz et al. [159]	Yes	Yes	Yes	Yes	Yes	Yes	Yes	Yes	Yes	Yes
Aranha et al. [142]	Yes	Yes	Yes	Yes	Yes	Yes	Yes	No	NR	Yes
Kessler et al. [184]	Yes	NR	NR	NR	NR	NR	NR	NR	NR	Yes
Kim et al. [182]	Yes	Yes	Yes	Yes	Yes	Yes	Yes	N	Yes	Yes
Pala et al. [178]	Yes	No	No	No	Yes	No	Yes	No	No	Yes
Alqurashi et al. [179]	Yes	Yes	Yes	Yes	Yes	Yes	Yes	Yes	No	No
Carvalho-Peixoto et al. [180]	Yes	No	Yes	No	No	No	Yes	No	No	No
Gale et al. [181]	Yes	Yes	Yes	Yes	Yes	Yes	Yes	Yes	Yes	Yes
Udani et al. [183]	Yes	No	No	No	Yes	Yes	Yes	No	Yes	Yes

Abbreviations: NR, not reported.

## Data Availability

Not applicable.

## Supplementary Materials

The following supporting information can be downloaded at: https://www.mdpi.com/article/10.3390/nu15040989/s1, Table S1: Descriptive results of the biases found in the included animal studies following SYRCLE’S guidelines.
