# Supplementary material for: Açaí (Euterpe oleracea Mart.) in Health and Disease: A Critical Review
Source: Nutrients. 2023 Feb 16;15(4):989. doi: 10.3390/nu15040989 (PMC9965320; doi:10.3390/nu15040989)
Supplement: Supplementary file 1 [file nutrients-15-00989-s001.zip › nutrients-2219285-supplementary.pdf]

Table - Lucas Fornari Laurindo - Online Supplementary Material

Table S1. Descriptive results of the biases found in the included animal studies following SYRCLE'S guidelines (Hooijmans et al., 2014).

Properties most studied	Study	Sequence generation	Baseline characteristics	Allocation concealment	Random housing	Binding (intervention)	Random outcome assessment	Blinding (outcome)	Incomplete outcome data	Selective outcome reporting	Other sources of bias
Antioxidant	(Barbosa et al., 2021)	No	Yes	Yes	Unclear	No	Unclear	Unclear	Yes	Yes	Yes
	(Alegre et al., 2020)	No	Yes	Yes	Unclear	Unclear	Unclear	Unclear	Yes	Yes	Yes
	(Nascimento et al., 2016)	Yes	Yes	Yes	Unclear	No	Unclear	No	Yes	Yes	Yes
	(Guerra et al., 2011)	Unclear	Yes	Yes	No	Unclear	No	No	Yes	Yes	No
Anti-inflammatory	(Favacho et al., 2011)	Unclear	Yes	Yes	Unclear	Unclear	No	Unclear	Yes	Yes	No
	(Moura et al., 2012)	Unclear	Yes	Yes	Unclear	Unclear	Unclear	Yes	Yes	Yes	Yes
Antimicrobial	(Ferreira et al., 2019)	Yes	Unclear	Unclear	Unclear	Unclear	Unclear	Unclear	Yes	Unclear	No
Gastroprotective	(Monteiro et al., 2021)	Unclear	Yes	Yes	Unclear	Unclear	No	Unclear	Yes	Yes	Yes
	(Cury et al., 2020)	Unclear	Yes	Yes	Unclear	Unclear	No	Unclear	Yes	Yes	Yes

Neuroprotective	(Souza et al., 2015)	Yes	Yes	Yes	Unclear	Unclear	Unclear	Unclear	Yes	Yes	Yes
	(Bem et al., 2020)	Unclear	Yes	Yes	Unclear	Yes	No	Yes	Yes	Yes	Yes
	(Yildirim et al., 2020)	Unclear	Yes	Yes	Unclear	Unclear	Unclear	Unclear	Yes	Yes	Yes
	(Souza et al., 2016)	Unclear	Yes	Yes	Unclear	Unclear	Unclear	Unclear	Yes	Yes	Yes
	(Carey et al., 2017)	Unclear	Yes	Yes	Unclear	No	Unclear	No	Yes	Yes	Yes
	(Souza et al., 2015)	Unclear	Yes	Yes	Unclear	No	Unclear	Unclear	Yes	Yes	Yes
	(Spada et al., 2009)	Unclear	Yes	Yes	Unclear	No	No	No	Yes	Yes	No
	(Poulose et al., 2017)	Unclear	Yes	Yes	Unclear	No	Unclear	No	Yes	Yes	Yes
	(Souza et al., 2019)	No	Yes	Yes	Unclear	Unclear	No	Unclear	Yes	Yes	Yes
Antilipidemic	(Faria et al., 2017)	Yes	Yes	Yes	Unclear	No	Unclear	Unclear	Yes	Yes	Yes
	(Souza et al., 2017)	Yes	Yes	Yes	Unclear	No	Unclear	No	Yes	Yes	Yes

	(Souza et al., 2012)	Yes	Yes	Yes	Unclear	No	No	Unclear	Yes	Yes	Yes
	(Souza et al., 2010)	Unclear	Yes	Yes	Unclear	No	Unclear	No	Yes	Yes	Yes
	(Silva et al., 2018)	Yes	Yes	Yes	Unclear	Unclear	No	Unclear	Yes	Yes	No
	(Feio et al., 2012)	Yes	Yes	Yes	Unclear	Unclear	Unclear	Yes	Yes	Yes	No
Hepatoprotective	(Barbosa et al., 2020)	Unclear	Yes	Yes	Unclear	Unclear	Unclear	Unclear	Yes	Yes	Yes
	(Carvalho et al., 2018)	Yes	Unclear	Unclear	Unclear	Yes	Unclear	Yes	Yes	Yes	Yes
	(Zhou et al., 2018)	Unclear	Yes	Yes	Unclear	No	Unclear	No	Yes	Yes	Yes
	(Bem, et al., 2018)	Yes	Yes	Yes	Unclear	No	Yes	Unclear	Yes	Yes	Yes
	(Pereira et al., 2016)	Unclear	Yes	Yes	Unclear	No	Unclear	Yes	Yes	Yes	Yes
	(Carvalho et al., 2019)	Unclear	Yes	Yes	Unclear	Unclear	Yes	Unclear	Yes	Yes	Yes
	(Guerra et al., 2015)	No	Yes	Yes	Unclear	Unclear	Yes	Unclear	Yes	Yes	Yes

	(Oliveira et al., 2015)	Yes	Yes	Yes	Unclear	Unclear	No	Unclear	Yes	Yes	Yes
Antidiabetic	(Bem, et al., 2018)	Yes	Yes	Yes	Unclear	No	Unclear	Unclear	Yes	Yes	Yes
Anticancer	(Fragoso et al., 2018)	Yes	Unclear	Unclear	Unclear	No	No	Yes	Yes	Yes	Yes
	(Perini, et al., 2018)	Yes	Unclear	Unclear	Unclear	No	Unclear	Yes	Yes	Yes	Yes
	(Fragoso et al., 2013)	Yes	Yes	Yes	No	Unclear	No	Unclear	Yes	Yes	Yes
	(Choi et al., 2017)	Unclear	Yes	Yes	No	Unclear	No	Yes	Yes	Yes	Yes
	(Romualdo et al., 2015)	Yes	Yes	Yes	Unclear	Unclear	Unclear	Yes	Yes	Yes	Yes
	(Monge-Fuentes et al., 2017)	No	Unclear	Yes	Unclear	Unclear	Unclear	Unclear	Yes	Yes	No
	(Stoner et al., 2010)	Yes	Yes	Yes	Yes	Unclear	Unclear	Unclear	Yes	Yes	Yes
Analgesic	(Sudo et al., 2015)	Unclear	Yes	Yes	Unclear	No	No	No	Yes	Yes	Yes
Cardiovascular protection	(Figueiredo et al., 2022)	No	Yes	Yes	Unclear	Unclear	No	Unclear	Yes	Yes	Yes

	(Vilhena et al., 2021)	Unclear	Yes	Yes	Unclear	Unclear	No	Unclear	Yes	Yes	Yes
	(Lavorato et al., 2021)	Yes	Yes	Yes	Unclear	No	Unclear	Unclear	Yes	Yes	Yes
	(Pontes et al., 2021)	No	Yes	Yes	Unclear	No	Unclear	No	Yes	Yes	Yes
	(Mathias et al., 2019)	Unclear	Yes	Yes	Unclear	Unclear	Unclear	Yes	Yes	Yes	Yes
	(Zapata-Sudo et al., 2014)	Unclear	Yes	Unclear	Unclear	No	Unclear	Unclear	Yes	Yes	Yes
	(Viviane Cordeiro et al., 2015)	Yes	Yes	Yes	Unclear	Unclear	Unclear	Unclear	Yes	Yes	No
Renoprotective	(Costa et al., 2017)	Unclear	Unclear	Unclear	Unclear	Unclear	Unclear	Unclear	Yes	Yes	Yes
	(Silva et al., 2018)	Unclear	Yes	Yes	Unclear	Unclear	Unclear	No	Yes	Yes	No
	(Unis, 2015)	Unclear	Yes	Yes	Unclear	Unclear	Unclear	Yes	Yes	Yes	No
	(El Morsy et al., 2015)	Yes	Yes	Yes	Unclear	Unclear	Unclear	No	Yes	Yes	Yes
Wound-healing	(Kang et al., 2017)	Yes	Yes	Yes	Unclear	Unclear	No	Unclear	Yes	Yes	Yes

	(Kang & Kim, 2018)	Yes	Yes	Yes	Unclear	No	Unclear	Unclear	Yes	Yes	Yes
Miscellaneous effects	(Muto et al., 2021)	Unclear	Yes	Yes	Unclear	Unclear	No	Unclear	Yes	Yes	Yes
	(Soares et al., 2020)	Yes	Yes	Yes	Unclear	Unclear	No	Unclear	Yes	Yes	Yes
	(D. E. Machado et al., 2016)	Yes	Yes	Yes	Unclear	Unclear	Unclear	Yes	Yes	Yes	Yes
	(Brasil et al., 2017)	Unclear	Yes	Unclear	Unclear	No	No	Unclear	Yes	Yes	Yes
	(Shibuya et al., 2020)	Unclear	Yes	Yes	Unclear	No	Unclear	No	Yes	Yes	Yes

References:

Hooijmans CR, Rovers MM, de Vries RBM, Leenaars M, Ritskes-Hoitinga M, Langendam MW. SYRCLE's risk of bias tool for animal studies. BMC Med Res Methodol 2014;14:43-. doi: 10.1186/1471-2288-14-43.
